# The Phytohormone Ethylene Enhances Cellulose Production, Regulates CRP/FNR_Kx_ Transcription and Causes Differential Gene Expression within the Bacterial Cellulose Synthesis Operon of *Komagataeibacter* (*Gluconacetobacter*) *xylinus* ATCC 53582

**DOI:** 10.3389/fmicb.2015.01459

**Published:** 2015-12-22

**Authors:** Richard V. Augimeri, Janice L. Strap

**Affiliations:** Molecular Microbial Biochemistry Laboratory, Faculty of Science, University of Ontario Institute of Technology, OshawaON, Canada

**Keywords:** ethylene, *Komagataeibacter* (*Gluconacetobacter*) *xylinus*, bacterial cellulose, CRP/FNR, indole-3-acetic acid (IAA), abscisic acid (ABA), plant-microbe interaction, fruit-bacteria interaction

## Abstract

*Komagataeibacter* (formerly *Gluconacetobacter*) *xylinus* ATCC 53582 is a plant-associated model organism for bacterial cellulose (BC) biosynthesis. This bacterium inhabits the carposphere where it interacts with fruit through the bi-directional transfer of phytohormones. The majority of research regarding *K. xylinus* has been focused on identifying and characterizing structural and regulatory factors that control BC biosynthesis, but its ecophysiology has been generally overlooked. Ethylene is a phytohormone that regulates plant development in a variety of ways, but is most commonly known for its positive role on fruit ripening. In this study, we utilized ethephon (2-chloroethylphosphonic acid) to produce *in situ* ethylene to investigate the effects of this phytohormone on BC production and the expression of genes known to be involved in *K. xylinus* BC biosynthesis (*bcsA, bcsB, bcsC, bcsD, cmcAx, ccpAx and bglAx*). Using pellicle assays and reverse transcription quantitative polymerase chain reaction (RT-qPCR), we demonstrate that ethephon-derived ethylene enhances BC directly in *K. xylinus* by up-regulating the expression of *bcsA* and *bcsB*, and indirectly though the up-regulation of *cmcAx, ccpAx*, and *bglAx*. We confirm that IAA directly decreases BC biosynthesis by showing that IAA down-regulates *bcsA* expression. Similarly, we confirm that ABA indirectly influences BC biosynthesis by showing it does not affect the expression of *bcs* operon genes. In addition, we are the first to report the ethylene and indole-3-acetic acid (IAA) induced differential expression of genes within the bacterial cellulose synthesis (*bcs*) operon. Using bioinformatics we have identified a novel phytohormone-regulated CRP/FNR_Kx_ transcription factor and provide evidence that it influences BC biosynthesis in *K. xylinus*. Lastly, utilizing current and previous data, we propose a model for the phytohormone-mediated fruit-bacteria interactions that *K. xylinus* experiences in nature.

## Introduction

*Komagataeibacter* (formerly *Gluconacetobacter*) *xylinus* ATCC 53582 is an acetic acid bacterium (Yamada et al., 2012; Mamlouk and Gullo, 2013) studied for its ability to synthesize and secrete large quantities of crystalline cellulose at the air-liquid interface of static cultures (Schramm and Hestrin, 1954). Bacterial cellulose (BC) produced by *K. xylinus* is of higher purity compared to plant cellulose since it is devoid of hemicellulose, pectin and lignin (Schramm and Hestrin, 1954; Kudlicka and Brown, 1996), allowing for a highly ordered, crystalline, and recalcitrant cellulose I matrix.

*Komagataeibacter xylinus* and related species can be isolated from fruit (Park et al., 2003; Dellaglio et al., 2005; Jahan et al., 2012; Neera et al., 2015) and spoiled wine (Bartowsky and Henschke, 2008). These are sugar-rich environments that provide ideal conditions for *K. xylinus* growth and BC biosynthesis. In the environment, *K. xylinus* synthesizes a BC biofilm to facilitate adherence and subsequent colonization of its fruit substrate. A biofilm is defined as a surface-associated bacterial community embedded within an extracellular matrix consisting of polysaccharides, proteins and extracellular DNA (Geesey et al., 1978; Costerton et al., 1995; Flemming and Wingender, 2010). The BC matrix also provides protection from environmental stresses and provides a competitive advantage over other microorganisms (Williams and Cannon, 1989). This phenomenon is also observed with *Enterobacter amnigenus* GH-1, which synthesizes BC to adhere to fruits and abiotic materials (Kim et al., 2006; Hungund and Gupta, 2010). Numerous other bacteria also synthesize biofilms containing BC and other polysaccharides to facilitate interactions with plants and animals. The role of BC in facilitating the diverse environmental interactions of various biofilm-producing bacteria has recently been reviewed (Augimeri et al., 2015).

**FIGURE 1:**
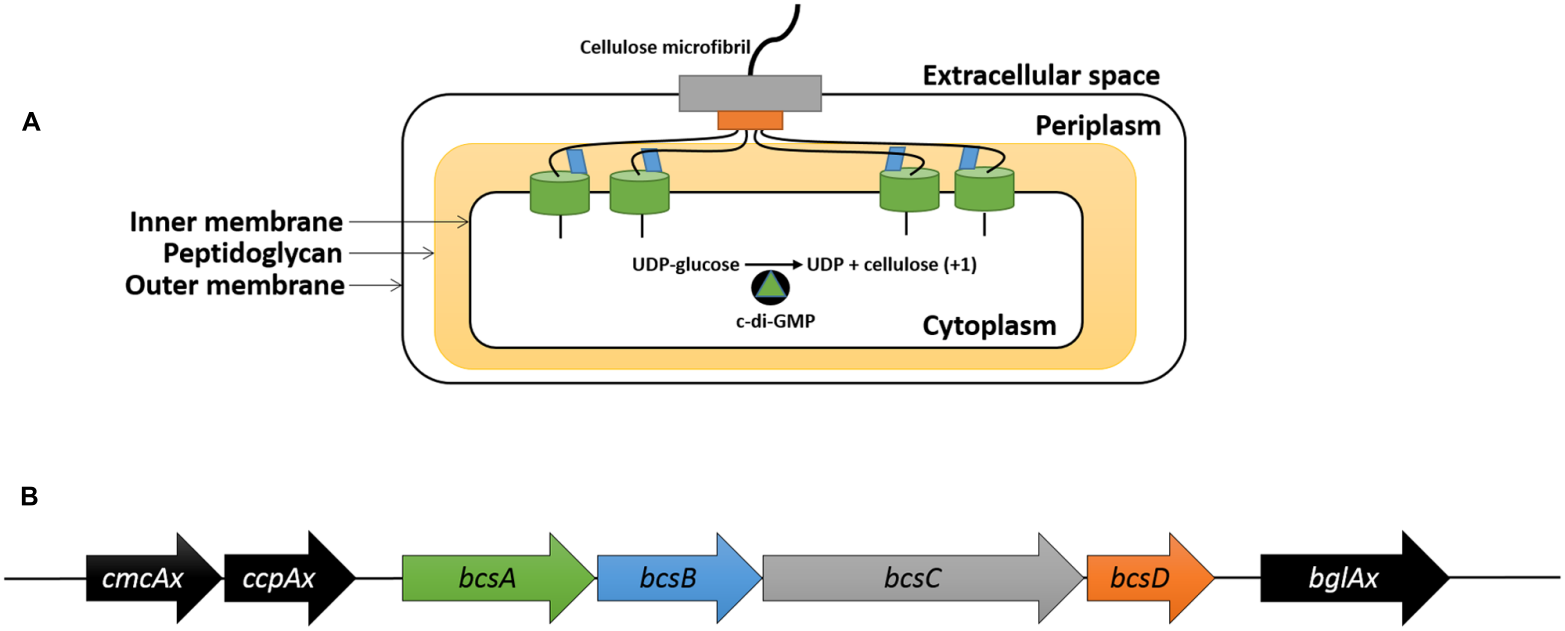
**Structural and genetic organization of the bacterial cellulose synthesis complex and the *bcs* operon.** BcsA (green), activated by c-di-GMP, adds a glucose unit to the cellulose chain using UDP-glucose as a substrate in the cytoplasm; BcsB (blue) guides the glucan chain through the periplasm; BcsD (orange) crystallizes four glucan chains in the periplasm; BcsC (gray) exports the BC microfibril into the extracellular space **(A)**. The genetic organization of the *bcs* operon that encodes the bacterial cellulose synthesis complex and the genes up- and downstream **(B)**. The genes in the *bcs* operon **(B)** are color coordinated with their protein products **(A)**.

Bacterial cellulose is synthesized on the cytoplasmic side of the inner membrane (Bureau and Brown, 1987) and is subsequently transported through the periplasmic space before it is released into the extracellular environment (**Figure 1A**; Morgan et al., 2013). BC synthesis terminal complexes are found along the longitudinal axis of the rod-shaped *K. xylinus* cell (Brown et al., 1976; Kimura et al., 2001; Sunagawa et al., 2013) and are responsible for the synthesis and export of BC. This arrangement allows adjacent glucan chains to be hydrogen bonded as they are being exported, and thus couples elongation, translocation, and crystallization of BC (Morgan et al., 2013).

The BC synthesis terminal complexes in *K. xylinus* ATCC 53582 consist of four protein subunits (BcsA, BcsB, BcsC, and BcsD) that are encoded within the BC synthesis (*bcsABCD*) operon (**Figure 1B**). The BcsA subunit, located on the cytoplasmic face of the inner membrane (Omadjela et al., 2013) possesses a catalytic β-1,4-glycosyltransferase domain containing the DDDQ(Q/R)XRW motif (Richmond and Somerville, 2000) responsible for polymerizing monomers of uridine diphosphoglucose (UDP-glucose) into β-1,4-glucan chains of cellulose (**Figure 1A**; Lin et al., 1990). The activity of the catalytic domain is regulated at the C-terminal PilZ domain (Fujiwara et al., 2013; Omadjela et al., 2013; Morgan et al., 2014) by the allosteric activator of BC synthesis, bis-(3′→5′)-cyclic diguanylate (c-di-GMP; Ross et al., 1987). The levels of c-di-GMP are modulated by three cyclic diguanylate (*cdg*) operons (Tal et al., 1998) that encode diguanylate cyclases (DGCs) and phosphodiesterases (PDEs) that synthesize (Qi et al., 2009) and degrade (Chang et al., 2001) c-di-GMP, respectively. Per-Arnt-Sim (PAS) sensory domains on these enzymes control the catalytic domains, c-di-GMP levels and thus BC biosynthesis, by perceiving oxygen levels and cellular redox status (Chang et al., 2001; Qi et al., 2009; Henry and Crosson, 2011). BcsB binds to BcsA in the periplasm by a single C-terminal transmembrane helix (Morgan et al., 2013), where it stabilizes BcsA and guides glucan chains through the periplasmic space using two carbohydrate-binding domains (CBD1 and CBD2; **Figure 1A**).

Extrusion of BC from the periplasm to the extracellular environment is believed to be facilitated through the action of BcsC (**Figure 1A**), which is predicted to form a pore in the outer membrane of *K. xylinus* based on its structure. Consistent with the view that BcsC is an outer membrane porin, is the observation that BcsC is essential for *in vivo*, but not *in vitro* BC biosynthesis (Saxena et al., 1994).

Crystallization of BC is achieved through the action of BcsD, a cylindrical octameric periplasmic protein (Iyer et al., 2011) that contains four spiral channels that facilitates hydrogen bonding of four glucan chains during export through BcsC (**Figure 1A**; Hu et al., 2010). In *Komagataeibacter hansenii* ATCC 23769, *bcsD* is regulated independently of the other *bcs* genes (Deng et al., 2013), as a 321 base-pair untranslated region (UTR) separates *bcsC* and *bcsD*, leaving room for a functional promoter (Deng et al., 2013). The coding regions of *bcsC* and *bcsD* in *K. xylinus* have a 1 base-pair overlap, suggesting that *bcsD* is regulated from the same promoter as the rest of the *bcs* genes in this strain.

Located upstream of the *bcs* operon, is *cmcAx* (**Figure 1B**), which encodes an endo-β-1,4-glucanase that has cellulose-hydrolyzing activity *in vitro* on cellopentose or longer oligosaccharide substrates (Kawano et al., 2002a). In small amounts, exogenous CmcAx enhances BC production of *K. xylinus* (Kawano et al., 2002a); while endogenous overexpression of *cmcAx* increases BC yield (Kawano et al., 2002a). This suggests that the cellulose hydrolyzing activity of CmcAx may exert a regulatory effect on BC biosynthesis.

In the same upstream operon as *cmcAx*, is *ccp*Ax (**Figure 1B**), which encodes the cellulose-complementing protein (CcpAx) that is required for *in vivo* BC biosynthesis (Deng et al., 2013; Standal et al., 1994), particularly its crystallization (Nakai et al., 2002). CcpAx is co-localized with BcsD longitudinally on the extracellular side of the cell membrane, suggesting that CcpAx is a critical part of the BC synthesis terminal complex (Sunagawa et al., 2013). Since this protein is of low molecular weight with predicted secondary structures rich in α-helices, CcpAx may facilitate protein-protein interactions for the spatial assembly of BC synthesis complexes (Sunagawa et al., 2013). Disruption of *ccpAx* results in a significant reduction in the levels of BcsB and BcsC (Deng et al., 2013), indicating CcpAx also plays a regulatory role in BC biosynthesis.

Downstream of the BC synthesis operon is *bglAx* (**Figure 1B**), which encodes a β-glucosidase (BglAx; Tajima et al., 2001). This monomeric enzyme is secreted (Tahara et al., 1998) and has the ability to hydrolyze oligosaccharides larger than three residues into single β-D-glucose units (Tahara et al., 1998; Tajima et al., 2001). In *K. hansenii* ATCC 23769, the expression of *bglAx* is transcriptionally regulated by CRP/FNR_Kh_, a cyclic-AMP receptor/fumarate nitrate reductase protein (Deng et al., 2013). Transposon insertion into *crp/fnr_Kh_* (GXY_00863) completely abolished production of BC and BglAx, providing evidence that CRP/FNR_Kh_ controls BC biosynthesis at the transcriptional level. Since *bglAx* deletion results in reduced BC synthesis but not the absence of BC production observed with *crp/fnr_Kh_* deletion, it is probable that CRP/FNR_Kh_ controls the expression of additional genes that are essential for BC biosynthesis (Deng et al., 2013).

The CRP/FNR family of transcription factors are ubiquitous in bacteria (Matsui et al., 2013) and regulate processes that are critical to bacterial growth and survival. Some of these processes include catabolite repression, aerobic growth, nitrogen fixation, oxidative stress responses, stationary phase survival, arginine catabolism, and pathogenicity (Körner et al., 2003).

*Komagataeibacter xylinus* is believed to participate in various fruit-bacteria interactions when inhabiting the carposphere. We previously showed that *K. xylinus* ATCC 53582 endogenously synthesized and secreted the phytohormones, zeatin, gibberellic acid (GA_3_), and abscisic acid (ABA; Qureshi et al., 2013), all of which are involved in fruit growth and ripening (McAtee et al., 2013). Unlike *Gluconacetobacter diazotrophicus* (Lee and Kennedy, 2000), *K. xylinus* does not produce indole-3-acetic acid (IAA; Qureshi et al., 2013), a phytohormone known to inhibit fruit ripening and senescence when applied exogenously to plants (Davies et al., 1997). *K. xylinus* cultures grown in the presence of exogenous zeatin, GA_3_, and ABA grew faster and had greater BC yields than untreated cultures (Qureshi et al., 2013). Exogenous IAA, however, increased growth but decreased the BC yield (Qureshi et al., 2013). The effect of IAA on BC production is therefore direct, while ABA, zeatin, and GA_3_ indirectly increase BC yield due to an enhanced growth rate. These results led us to investigate ethylene, a fruit ripening phytohormone directly affected by the concentrations of IAA and ABA in plants, for its effects on BC biosynthesis.

Ethylene is a gaseous phytohormone important to many aspects of plant growth and development, including having a positive role on ripening, senescence and rotting of climacteric fruit (Lieberman, 1979). Decreased IAA levels with a concomitant increase in ABA concentration during fruit growth, triggers the biosynthesis of ethylene (Zhang et al., 2009) which induces expression of genes whose protein products are required for the enzymatic degradation of fruit polysaccharides into soluble monosaccharides (Zegzouti et al., 1999). Numerous studies have investigated the effect of bacterially produced ethylene on plants (Weingart and Völksch, 1997; Baca and Elmerich, 2007), as well as the effect of lowering plant-produced ethylene levels by bacterially produced 1-aminocyclopropane-1-carboxylate (ACC) deaminase enzymes (Glick, 2005). However, there is a paucity of literature regarding the effect of plant-produced ethylene on bacteria. This may be because ethylene is a gas and thus difficult to control in a laboratory setting without specialized equipment. Ethephon (2-chloroethylphosphonic acid) is an ethylene-releasing compound that produces *in situ* ethylene at a 1:1 molar ratio above pH 3.5 (Zhang and Wen, 2010). Base-catalyzed chemical degradation of ethephon results in the production of ethylene, chloride, and phosphate through a first-order reaction (Warner and Leopold, 1969; Biddle et al., 1976; Klein et al., 1979). The rate of ethephon decomposition is positively correlated with pH and temperature (Biddle et al., 1976; Klein et al., 1979). Like ethylene, numerous studies have shown that application of ethephon induces and accelerates ripening of various fruits (Jolliffe, 1975; Hardie et al., 1981; Szyjewicz et al., 1984; Ban et al., 2007; Dhillon and Mahajan, 2011; Zhang et al., 2012; Dhall and Singh, 2013), allowing ethephon to be used in agriculture as a chemical replacement for gaseous ethylene during pre- and post-harvest fruit ripening (Singh and Janes, 2001; Khorshidi and Davarynejad, 2010; Gill et al., 2014).

In plants, ethylene binds to ethylene-binding domains (EBDs) that belong to a family of ethylene receptors that subsequently activate intracellular signal cascades that leads to ethylene-dependent phenotypes (Lacey and Binder, 2014). A bioinformatics study has shown that EBDs are common in plants and cyanobacteria, occur in fungi and green algae, but are generally absent in archeabacteria and eubacteria (Wang et al., 2006). However, Kim et al. (2007) demonstrated that various *Pseudomonas* species, including plant-associated *P. syringae* and *P. putida*, were chemotactic towards ethylene and that a mutant with the deletion of a methyl-accepting chemotaxis protein (MCP) gene (*che*R) was unable to respond to ethylene, suggesting that ethylene chemotaxis involved one or more MCPs. The CheR protein contains two domains: the CheR domain, which is involved in chemotaxis signaling and sensing environmental signals and an AdoMet_MTase domain that use *S*-adenosyl-L-methionine as a substrate for methyl group transfers (i e., methyltransferase) establishing that bacteria have the ability to respond to exogenous ethylene.

Protein alignments comparing CheR of *P. aeruginosa* PAO1 to the *K. xylinus* E25 genome reveals that *K. xylinus* contains a putitive MCP (27% identities; *E* = 1e^-27^; 90% query coverage; Gene: H845_2135). A search of the *K. xylinus* E25 genome showed that this putitive MCP is co-located on the chromosome with a methyl-accepting sensory transducer gene (H845_2133), various genes involved in chemotaxis, such as *cheW* (H845_2134 and H845_2136), *cheA* (H845_2132), and *cheB* (H845_2131), a histidine kinase signal transduction response regulator (H845_2130), and a PAS/PAC hybrid histidine kinase sensor (H845_2129). The *cheB* gene encodes a methyltransferase, *cheW* encodes a small regulator protein unique to bacterial chemotaxis, and *cheA* encodes a histidine kinase. In addition to its role in chemotaxis, *cheA* affects biofilm formation in *Pseudomonas* (Tremaroli et al., 2011).

In this study, we used ethephon to analyze the effects of exogenous ethylene on *K. xylinus* ATCC 53582 growth, BC production and pellicle properties. Release of ethylene from ethephon decomposition in a pH 7 Schramm-Hestrin (SH) medium was verified using the *Arabidopsis thaliana* triple response assay. Ethylene increased the crystallinity of *K. xylinus* BC pellicles, which resulted in decreased pellicle hydration. *K. xylinus* grown in the presence of ethylene on solid medium and in broth cultures produced more BC than controls, while the growth rate in agitated broth cultures was not affected, indicating that the positive effect of ethylene on BC biosynthesis was direct. These results suggest that ethylene produced by ripening fruit may aid in *K. xylinus* fruit colonization by enhancing the production and recalcitrance of its BC, thereby providing an advantage against competing microorganisms in nature. Reverse transcription quantitative polymerase chain reaction (RT-qPCR) assays revealed that ethylene, IAA and ABA affect the expression of genes known to be involved in *K. xylinus* BC biosynthesis. We report for the first time, the differential expression of genes within the *bcs* operon, as well as the identification of a phytohormone-regulated CRP/FNR transcription factor gene in *K. xylinus* (*crp/fnr_Kx_*). In addition, we are the first to use ethephon to study the effects of ethylene on bacteria. This study elaborates on the putative fruit-bacteria interactions of *K. xylinus* and gives new insights into the transcriptional regulation of the *bcs* operon.

## Materials and Methods

### Chemicals

Stocks of ethephon (2-chloroethylphosphonic acid; Sigma), NaCl (BioBasic), and NaH_2_PO_4_⋅H_2_O (BioBasic) were dissolved in ultra-pure water (pH 2.5), while indole-3-acetic acid (IAA; BioShop) and abscisic acid (ABA; BioShop) were prepared in 100% dimethyl sulfoxide (DMSO; BioBasic). All stock solutions were filter-sterilized and frozen at -20^o^C until used.

### Bacteria and Culture Conditions

*Komagataeibacter xylinus* ATCC 53582 was maintained as frozen glycerol stocks at -80°C. All starter cultures were grown by inoculating a single rough colony of *K. xylinus* from an SH agar plate streaked from glycerol stock, in 5 mL of SH broth (pH 5) supplemented with 0.2% (v/v) filter-sterilized cellulase. Cultures were grown in triplicate and incubated at 30°C with agitation at 150 rpm until the cells reached an OD_600_ of 0.3-0.4. Cultures were harvested by centrifugation, washed twice and suspended in sterile 0.85% (w/v) NaCl and quantified with a Petroff-Hauser counting chamber. All experiments employed an inoculum level of 10^5^ cells/mL, incubation at 30°C, and shaking at 150 rpm if grown with agitation.

### Triple Response Assay

Ethylene production from ethephon decomposition on SH medium (pH 7) was verified using the *Arabidopsis thaliana* triple response assay (Guzmán and Ecker, 1990). Seeds of *A. thaliana* ecotype Columbia were surface-sterilized in a sealed plastic container using chlorine gas produced from the addition of 3 mL concentrated HCl into 100 mL of bleach. The assay was performed using glass petri dishes that were divided into four quadrants. Growth medium for seeds was added in the quadrants adjacent to those containing SH agar at pH 7 (Supplementary Figure S1). Negative controls consisted of sterile seeds plated on 1X Murashige and Skoog salts medium (MS; pH 6.0; 0.8% agar) containing 1% (w/v) sucrose. Positive controls consisted of seeds plated on the MS-sucrose medium supplemented with 10 μM 1-aminocyclopropane carboxylic acid (ACC), the precursor for ethylene biosynthesis in plants. Ethephon (1 mM) was spread on pH 7 SH agar (1.5%) with seeds plated on the adjacent MS-sucrose medium. Plates were sealed with Parafilm, wrapped in foil and incubated in the dark for 4 days at 4°C before being exposed to light for 2 h. Stratified seeds were germinated in the dark for 72 h at 23°C. The experiment was performed with three biological replicates. Seedlings were photographed using a Cannon digital SLR camera or a USB 2.0 USB Digital Microscope (Plugable Technologies). The hypocotyl length of 60 seedlings per biological replicate (180 seedlings per treatment) was measured using ImageJ software (Schneider et al., 2012). Statistics were performed using a one-way ANOVA with a Tukey’s multiple comparison test. Differences were considered statistically significant if *p* < 0.05.

### Time-course pH Analysis

The pH change during growth of *K. xylinus* cultures in the presence of ethephon was assessed. Starter cultures were used to inoculate 150 mL of SH medium (pH 7) supplemented with 0.2 % (v/v) cellulase and were incubated with agitation for 14 days. Ethephon was tested at concentrations of 0.01, 0.1, and 1.0 mM, while the untreated control culture was supplemented with an equal volume of acidified ultra-pure water (pH 2.5). An identical experiment using phosphate and chloride as the test compounds was run in parallel. All flasks were covered with foil and sealed tightly with tape to prevent the escape of released ethylene. Each day, an aliquot of culture was removed and the pH was measured. Three biological replicates were tested with three technical replicates each. Statistical analysis was completed using a one-way ANOVA with Tukey’s multiple comparisons test. Differences were considered significant if *p* < 0.05.

### Minimum Inhibitory Concentration (MIC) Assay

Minimum inhibitory concentration assays to determine the lowest concentration of ethephon that prevented visible growth of *K. xylinus* were performed in sterile 96-well plates using the two-fold serial broth dilution MIC method (Witebsky et al., 1979). Ethephon was tested using a concentration range of 0.195-100 mM and was diluted in SH broth (pH 7). Wells were inoculated with *K. xylinus* starter cultures in a final volume of 150 μL. Growth controls lacking ethephon and sterile controls were included. Plates were sealed with Parafilm and incubated statically for 5 days. Clear wells were an indication of growth inhibition. To ensure the results were caused by ethylene and not by chloride or phosphate, the two by-products that result from ethephon decomposition, the same experiment was performed using phosphate and chloride as the test compounds. Three biological replicates were tested with one technical replicate each.

### Growth Kinetics

The effect of ethephon on the growth of agitated *K. xylinus* cultures was determined in 96-well plates that were inoculated, in triplicate, with *K. xylinus* starter cultures in a final volume of 200 μL of SH medium (pH 7) supplemented with 0.4% (v/v) cellulase. Three biological replicates and sterile controls, each with six technical replicates, were included for each treatment. Ethephon concentrations of 0.01, 0.1, and 1.0 mM, along with an untreated control plate that was supplemented with sterile ultra-pure water (pH 2.5), were all tested using separate plates. All unused wells contained sterile water. Two experiments were conducted; one in which ethephon or ultra-pure water (pH 2.5) was added only at the beginning of the experiment, while the second included the addition of ethephon or ultra-pure water (pH 2.5) every two days. All plates were sealed with Parafilm and incubated with agitation as described above. Optical density (OD) was recorded at 600 nm using a Bio-Rad xMark^TM^ Microplate Absorbance Spectrophotometer (Bio-Rad). Growth was followed for 335 h.

### Pellicle Assays and Analysis

*Komagataeibacter xylinus* pellicles grown in the presence of ethephon were characterized to determine the effect of ethylene on BC production under static conditions. Pellicle assays were conducted in sterile 24-well plates using a final well volume of 2 mL of SH broth (pH 7). Wells were inoculated with *K. xylinus* starter cultures using three biological replicates, each with six technical replicates. Ethephon stock solution was added to obtain final ethephon concentrations of 0.01, 0.1, and 1.0 mM. Each plate contained a row of sterile control wells that consisted of only medium and ethephon to control for contamination. All plates were sealed with Parafilm and incubated statically for 7 days. Untreated control plates, as well as phosphate-chloride (0.01, 0.1, and 1.0 mM) control plates were run in parallel. Each treatment was run in its own plate to prevent crossover of ethylene. Additionally, control and ethephon-treated plates were spatially separated by growing them in different incubators.

The thickness of *K. xylinus* pellicles was measured without the removal of water at the time of harvest. All pellicles, aligned with a ruler, were photographed from the side and measured at three different positions using ImageJ software (Schneider et al., 2012). These values were averaged to get one value for each technical replicate, which were averaged to obtain one value for each biological replicate.

Pellicle water-holding capacity and BC yield was determined by measuring the wet weights and dry weights, respectively. Pellicle wet weights were determined by removing pellicles from the plates and then holding them on paper towel for three seconds to remove excess medium before weighing. BC yield was determined by treating the pellicles with 0.1 N NaOH at 80°C for 20 min to lyse cells. Pellicles were neutralized by shaking in ultra-pure water for 24 h with two water changes and then dried in microcentrifuge tubes at 50°C to constant weight before being weighed for pellicle dry weight. Pellicle hydration was calculated by subtracting the dry weight from the wet weight.

The crystallinity index, CI(IR), of untreated *K. xylinus* pellicles as well as those formed in the presence of 0.01, 0.1, and 1.0 mM ethephon and phosphate-chloride, were assessed using Fourier-transform infrared spectroscopy (FTIR). Pellicles from each treatment that had been NaOH-treated, washed and dried were used for FTIR analysis on a Perkin Elmer Precisely Spectrum 100 FTIR spectrometer with a horizontal attenuated total reflectance sampling accessory. For each treatment, three technical replicates for each of the three biological replicates were analyzed using 32 scans with a resolution of 4 cm^-1^ in the range of 4000 to 650 cm^-1^. Background correction was performed prior to collecting sample data. CI(IR) was calculated using A_1437/_A_895_ as previously described (Czaja et al., 2004).

Values for technical replicates of pellicles were averaged to get one value for each biological replicate, which was used for statistical analysis. The means of ethephon-treated cultures were normalized to the mean of the control cultures and presented as the percent of the control. A one-way ANOVA with a Tukey’s multiple comparison test was used. Differences were considered statistically significant if *p* < 0.05.

### Colony Morphology

The effect of ethephon-derived ethylene on the morphology of *K. xylinus* colonies was assessed using an agar plate assay. Plates were made with 25 mL of SH agar (pH 7) and ethephon or phosphate-chloride were spread onto plates at concentrations of 0.01, 0.1, and 1.0 mM. Untreated and solvent control plates which consisted of no spreading and the spreading of ultra-pure water (pH 2.5), respectively, were also performed. Triplicate *K. xylinus* starter cultures were grown, washed and inoculated onto agar plates that were sealed with Parafilm and incubated statically for 5 days. Colonies were photographed with a USB 2.0 USB Digital Microscope (Plugable Technologies). All treatments included three biological replicates.

### Reverse-transcription Quantitative Polymerase Chain Reaction (RT-qPCR)

#### Growth Conditions

*Komagataeibacter xylinus* was grown in the presence of ethephon, ABA, and IAA to assess the effect these hormones had on the expression of genes involved with BC biosynthesis. For ethephon- and phosphate-chloride-treated cultures, triplicate *K. xylinus* starter cultures were grown, pooled, washed, and quantified. A 2 L Erlenmeyer flask, containing 1 L of SH broth (pH 5) with 0.2 % (v/v) cellulase was inoculated and incubated until an OD_600_ of 0.4-0.5 was reached. Cells were harvested by centrifugation, washed twice with room temperature SH broth (pH 7), and suspended in 1 L of SH broth (pH 7). This synchronized culture was then separated into 25 mL cultures, which were supplemented with 10 μM ethephon, 10 μM phosphate-chloride, or an equal volume of ultra-pure water (pH 2.5) for the control. These cultures were incubated for 24 h to allow for the decomposition of ethephon, and subsequent release of ethylene before being harvested. Flasks were covered with foil and tightly sealed with tape to trap released ethylene. Cultures treated with IAA and ABA were inoculated similarly to those treated with ethephon. After the OD_600_ reached 0.4-0.5, the culture was separated into triplicate 25 mL cultures and supplemented with IAA or ABA at a concentration of 10 μM. A control experiment consisting of DMSO-treated cultures was performed in parallel. Cultures were harvested 1 hour after being treated with hormone. At the time of harvest, for the ethephon, IAA, and ABA experiments, medium-free cell pellets were flash-frozen in liquid nitrogen and stored at -80^o^C until RNA extraction could be completed (no longer than 7 days). All treatments included three biological replicates.

### RNA Purification, Quality Control, and First-strand cDNA Synthesis

Flash-frozen cell pellets (10^9^ cells) were subject to total RNA extraction and purification using the Norgen Biotek RNA Purification Plus Kit following manufacturer instructions. Cells were not allowed to thaw prior to treatment with lysis buffer to ensure no change in mRNA levels occurred. Genomic DNA (gDNA) was removed by passing the crude RNA through a gDNA-removal column provided in the kit.

Quality control of RNA was completed on the same day of cDNA synthesis. RNA quality was determined by agarose gel electrophoresis. RNA concentration and purity was determined spectrophotometrically on a Cary 50 UV-Vis Spectrophotometer (Varian), by measuring the A_260_ and A_260_/A_280_ values, respectively. Only samples with A_260_/A_280_ values within 1.9-2.1 were used for cDNA synthesis. All samples were diluted to the same concentration using sterile diethylpyrocarbonate (DEPC)-treated water. In a reaction volume of 40 μL, 2 μg of RNA from each sample was converted to first-strand cDNA using the Bio-Rad iScript Select cDNA Synthesis Kit using random hexamer primers according to manufacturer instructions. Three samples were chosen at random from each experiment, and were subject to a mock-cDNA reaction that contained all components except reverse transcriptase, to assess RNA samples for gDNA contamination using RT-qPCR. All cDNA samples were stored at -20^o^C.

### Primer Design

RT-qPCR primers were designed and validated for eight genes involved in *K. xylinus* BC production (*bcsA, bcsB, bcsC, bcsD, cmcAx, ccpAx, bglAx*, and *crp*/*fnr_Kx_*; **Table 1**), and also two reference genes (*23SrRNA* and *gyrB*; **Table 1**). BlastN analysis comparing the nucleotide sequence of *crp/fnr_Kh_* (GXY_00863) to the *K. xylinus* E25 genome sequence (accession: CP004360.1) revealed the presence of a *crp/fnr_Kx_* gene (H845_3156) that is 79% similar (*E* = 4e^-175^, 97% query coverage) to *crp/fnr_Kh_*. BlastX analysis showed that the encoded protein sequences have 81% identity (*E* = 1e^-121^, 94% query coverage). This high level of similarity led us to hypothesize that CRP/FNR_Kx_ may regulate BC biosynthesis in *K. xylinus* ATCC 53582, similar to the results of Deng et al. (2013) that showed CRP/FNR_Kh_ regulates BC biosynthesis in *K. hansenii* ATCC 23769. Since the *K. xylinus* ATCC 53582 genome sequence is not published, primers for *23SrRNA* and *gyrB* were also designed using the nucleotide sequence of identical genes from other *K. xylinus* strains (Supplemental Table S1). End-point PCR was conducted using the *crp/fnr_Kx_, 23SrRNA* and *gyrB* primer sets and *K. xylinus* ATCC 53582 gDNA as the template to ensure the expected amplicons were produced. Primer specificity was assessed empirically by end-point PCR using *K. xylinus* ATCC 53582 gDNA as the template and by RT-qPCR melt-curve analysis. All primer sets were designed using Primer3Plus (Untergasser et al., 2007) to be 20-27 base-pairs in length, have a GC content of 45-55%, a melting temperature (*T*_m_) of 55-65°C, and to produce an amplicon of 90-300 base-pairs. Sequences were checked to be specific *in silico* using the Primer-Blast program^1^ to compare primer sequences to the genome sequences of various *K. xylinus* strains. The mfold web server^2^ was used to check primers and amplicons for potential secondary structures. Validated primer sequences are given (Supplemental Table S1).

**Table 1:** Function of proteins encoded by genes analyzed in this study.

Gene name	Encoded protein	Protein function
*gyrB*	DNA gyrase B subunit	DNA replication
*23SrRNA*	23S ribosomal RNA subunit	Structural component of ribosome
*bcsA*	BC synthase A subunit (BcsA)	Glycosyltransferase/BC synthesis
*bcsB*	BC synthase B subunit (BcsB)	Carbohydrate binding/BC chaperone
*bcsC*	BC synthase C subunit (BcsC)	Outer-membrane pore/BC export
*bcsD*	BC synthase D subunit (BcsD)	BC crystallization
*cmcAx*	Endo-β-1,4-glucanase (CmcAx)	BC hydrolysis/regulation
*ccpAx*	Cellulose-complimenting protein (CcpAx)	BC crystallization/regulation
*bglAx*	β-glucosidase (BglAx)	BC hydrolysis/regulation
*crp/fnr_Kx_*	Cyclic-AMP receptor protein/fumarate-nitrate reductase (CRP/FNR_Kx_)	Transcription factor

*See introduction for details.*

### RT-qPCR

The relative expression levels of these genes were determined after treatment with 10 μM ethephon, IAA, and ABA using the CFX Connect Real-Time PCR Detection System (Bio-Rad). RT-qPCR reactions (10 μL) included the SsoFast^TM^ EvaGreen^®^ Supermix (Bio-Rad), 300 or 500 nM primers (Supplemental Table S2), and 4 μL of the appropriate cDNA sample. Annealing temperature gradients were performed to empirically determine the optimal annealing temperature (*T*_a,opt._) for each primer set. The template for annealing temperature gradients consisted of aliquots of cDNA from each sample that were pooled and diluted 1/20 in 10 mM Tris-HCl (pH 8). The *T*_a,opt._ was chosen as the annealing temperature that resulted in the lowest *C*_t_ value and caused specific amplification as shown by melt-curve analysis. Standard curves were done to ensure the PCR amplification efficiency of each primer set at their optimal annealing temperature was between 90 and 110% (Taylor et al., 2010). The same pooled and 1/20-diluted cDNA were subject to a minimum of four serial dilutions and used as the template for standard curves. The fold-dilution depended on the expression level of each gene, as determined from annealing temperature gradients. The cDNA template concentration used for expression analysis (Supplemental Table S2) was determined by the linear dynamic range (LDR) of each primer set, as determined from the standard curves. Samples to be compared were diluted to the middle range of the LDR of each primer set. Every run included a no template control (NTC) containing 10 mM Tris-HCl (pH 8) as the template to check for reagent contamination and a no reverse-transcriptase (NRT) control containing the pooled mock cDNA reaction as the template to check for gDNA contamination. The qPCR reaction conditions were as follows: 2 min at 95.0°C, 40 cycles of 95.0°C for 5 s, the *T*_a,opt._ (Supplemental Table S2) for 15 s, and 72.0°C for 5 s. Melt-curve analysis was carried out at the end of every run with an initial denaturation at 95.0°C for 10 s, and then a temperature gradient from 65.0 to 95.0°C by steps of 0.5°C every 5 s. RT-qPCR plates (96-well) were loaded using the sample maximization strategy as previously described (Hellemans et al., 2007) by analyzing all samples with a particular gene on one plate. Each RT-qPCR was run in triplicate for three biological replicates per treatment.

### RT-qPCR Quality Control, Data Analysis, and Statistics

Quality control of raw Ct values was completed after every RT-qPCR run. Technical replicates were excluded if the standard deviation of their Ct values was over 0.2, but the mean of at least two technical replicates for each sample was used for comparison. Data analysis was carried out using the ΔΔC_t_ method (Livak and Schmittgen, 2001) employed in the Bio-Rad CFX Manager 3.1 Gene Study software using efficiency-corrected Ct values. Ct values from ethephon and phosphate-chloride RT-qPCR were made relative to Ct values obtained from the ultra-pure water (pH 2.5) control samples, while Ct values from IAA and ABA RT-qPCR were made relative to Ct values from their respective DMSO-treated controls. All RT-qPCR data was then normalized to the geometric mean of the efficiency-corrected expression data for the *23SrRNA* and *gyrB* reference genes. The stability of the reference genes was assessed using the geNorm algorithm (Vandesompele et al., 2002) within the Bio-Rad CFX Manager 3.1 Gene Study software, which uses a pair-wise comparison approach to calculate a gene stability value (*M*). Reference genes are considered stable if the *M* value is below 0.5 (Hellemans et al., 2007). Expression differences between hormone-treated and control samples were tested for statistical significance in the Bio-Rad CFX Manager 3.1 Gene Study software using a two-tailed, unpaired *t*-test. Differences were considered statistically significant if *p* < 0.05.

## Results

### Decomposition of Ethephon into Ethylene Occurs in SH Medium (pH 7)

**FIGURE 2:**
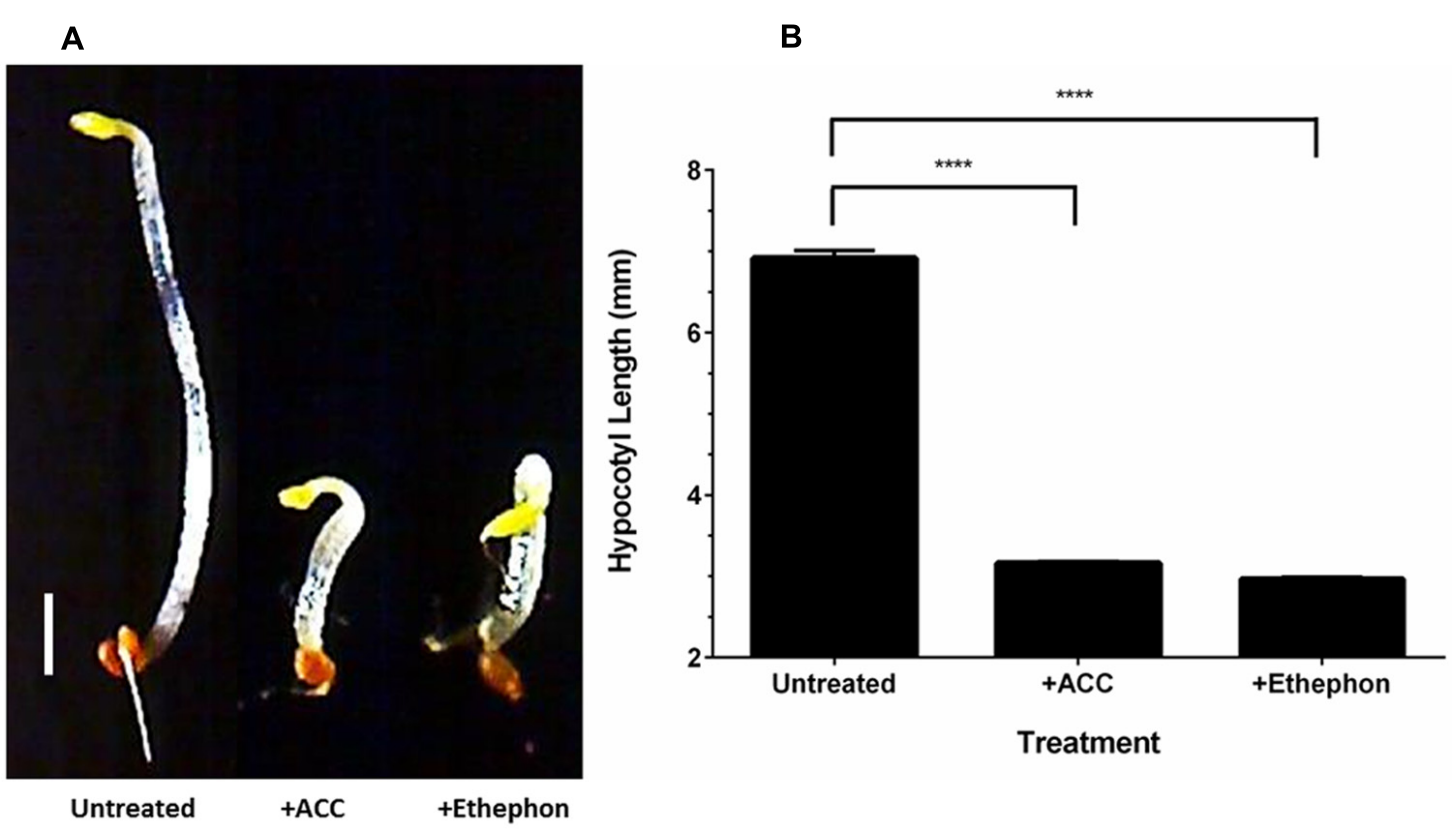
**Ethylene is released via ethephon decomposition in SH medium (pH 7).** Dark-grown *Arabidopsis thaliana* seedlings display the triple response phenotype (shorter and thicker hypocotyl with exaggerated apical hook) when grown in the presence of ACC and ethephon-derived ethylene compared to the untreated control **(A)**. The hypocotyl length of seedlings grown in the presence of ACC and ethephon-derived ethylene were significantly shorter than the untreated control **(B)**. Scale bar represents 1 mm. ^∗∗∗∗^*p* < 0.0001. Error bars show *SD* (*n* = 3).

The production of ethylene from ethephon decomposition on SH medium (pH 7) was verified using the triple response assay. When grown in the presence of ethylene, dark-grown *A. thaliana* seedlings display reduced hypocotyl elongation, increased hypocotyl thickness and an exaggerated apical hook (Guzmán and Ecker, 1990). Growing seedlings in medium containing ACC, the precursor for ethylene biosynthesis in plants, allows for the production of ethylene and induction of the triple response phenotype. Ethephon decomposition was performed on SH agar (pH 7) in a sectored petri dish containing *A. thaliana* seeds on MS-sucrose agar. The hypocotyl thickness was increased and the apical hook was exaggerated for seedlings grown in the presence of ACC and ethephon compared to the untreated control (**Figure 2A**). The apical hook of seedlings grown in the presence of ethephon-derived ethylene was more exaggerated than those grown with ACC. In addition, the hypocotyl length of seedlings grown in the presence of ACC and ethephon were significantly (*p* < 0.0001) shorter than the untreated control (**Figure 2B**). This result confirms that ethephon decomposition occurs in SH medium (pH 7) and that sufficient ethylene is released to induce a biological response.

### *K. xylinus* Culture pH Allows for Ethephon Decomposition

**FIGURE 3:**
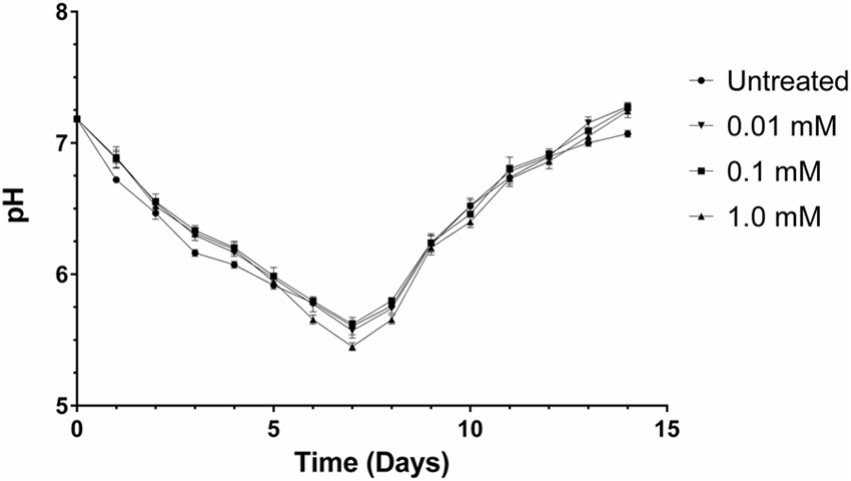
**The pH of *K. xylinus* cultures stays above 3.5, allowing for efficient decomposition of ethephon into ethylene.** *K. xylinus* was grown in SH medium (pH 7) that was supplemented with ethephon and 0.2% (v/v) cellulase. The change in culture pH was monitored for 14 days. Note that the y-axis begins at pH 5, and that the final pH of all ethephon-treated cultures is higher than the untreated control. Error bars show SD (*n* = 3).

*Komagataeibacter xylinus* is an acetic acid bacterium and is known to decrease the pH of its culture medium (Kawano et al., 2002b). Therefore, we performed a time-course pH analysis over 14 days to ensure the culture pH remained above 3.5 so that ethephon decomposition into ethylene would proceed unimpaired. The pH of all cultures decreased from pH 7 to approximately pH 5.5 for the first 7 days, wherein the pH increased back to pH 7 (**Figure 3**). Thus, acidification of the culture medium by *K. xylinus* would not have significantly affected ethephon decomposition and consequently, ethylene evolution. In addition, all cultures containing ethephon had a higher final pH than the untreated control culture (Supplementary Figure S2). The final pH of phosphate-chloride cultures were not different from the untreated control (data not shown), confirming the higher pH observed with ethephon treatment was due to the presence of ethylene.

### Ethylene is Relatively Non-toxic to *K. xylinus*

Minimum inhibitory concentration assays were performed to assess whether ethephon inhibited the growth of *K. xylinus*. Ethephon concentrations of 0.195 to 100 mM were tested in SH (pH 7). Concentrations equal to or greater than 50 mM inhibited growth of *K. xylinus* (data not shown). This effect was not observed when chloride and phosphate were tested at the same concentration, suggesting that ethephon-derived ethylene was responsible for *K. xylinus* growth inhibition. Growth inhibition was observed for very high, physiologically irrelevant levels of chloride-phosphate. In this case, we cannot rule out the possibility that the observed growth inhibition in the 100 mM chloride-phosphate treatment may be due to the Na^+^ counter-ion. Since NaCl and NaH_2_PO_4_⋅H_2_O were both added at 100 mM, the [Na^+^] would have been 200 mM, which could have prevented the growth of *K. xylinus* due to osmolarity effects.

### Ethylene does not Affect the Growth of *K. xylinus* in Agitated Broth Cultures

The effect of ethephon-derived ethylene on the growth of agitated *K. xylinus* broth cultures grown in SH medium (pH 7) was investigated. Since ethylene is a gas and the 96-well plates had to be opened to read the optical density, some ethylene was inevitably lost during this process. To determine if this loss was a factor, we either added ethephon only on the day of inoculation or on the day of inoculation and every two days thereafter. When compared to controls, no significant difference in growth was observed when ethephon was added to cultures at the time of inoculation (Supplementary Figure S3A) or when ethephon was added every 2 days (Supplementary Figure S3B). The same result was obtained when comparing the ethephon-treated cultures to phosphate-chloride controls. Therefore, ethylene does not influence *K. xylinus* growth in agitated culture.

### Ethylene Increases BC Yield and Decreases *K. xylinus* Pellicle Hydration Due to an Increase in Crystallinity

**FIGURE 4:**
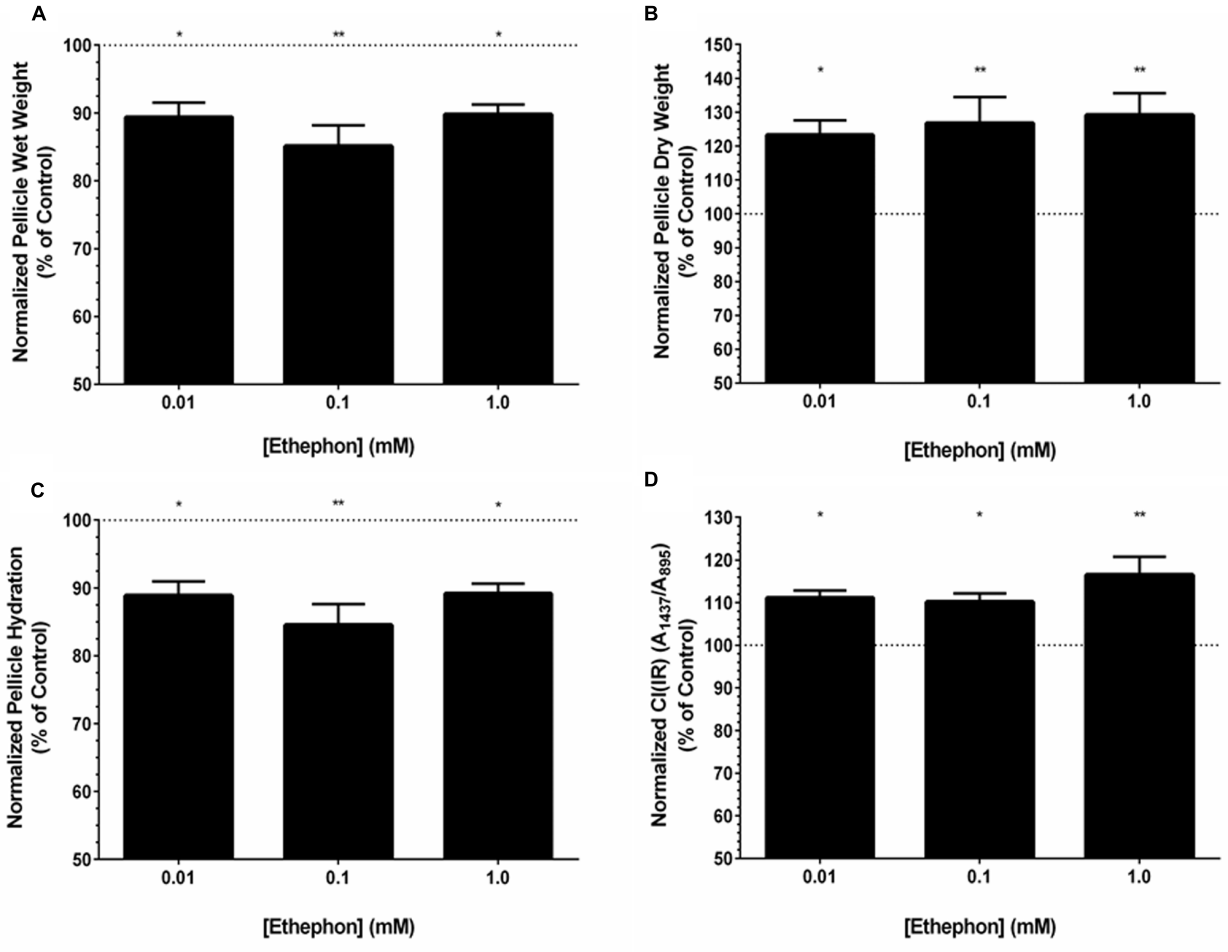
**Ethephon-derived ethylene influences the properties and yield of *K. xylinus* BC pellicles.** Cultures were grown statically in SH broth (pH 7) in 24-well plates, and incubated at 30°C for 7 days before pellicles were harvested and analyzed. Ethephon-derived ethylene decreases the wet weight **(A)**, increases the dry weight **(B)**, reduces pellicle hydration **(C)**, and increases the crystallinity **(D)** of *K. xylinus* pellicles compared to the untreated control. The different ethephon treatments were not significantly different from each other. Data was normalized to and expressed as percent of the untreated control. Note the y-axis begins at 50%. Error bars show *SD* (*n* = 3). ^∗^*p* < 0.05; ^∗∗^*p* < 0.01.

Pellicles formed by *K. xylinus* grown in the presence of ethephon were weighed at the time of harvest without the removal of water and after drying to assess their hydration. The wet weight of pellicles was affected by ethephon when cultured in SH medium at pH 7 (**Figure 4A**). The wet weight of pellicles produced in the presence of ethephon was decreased by 10% with 0.01 mM ethephon, 14% in the presence of 0.1 mM ethephon, and 9% with 1.0 mM ethephon, in comparison to the untreated control. The relationship between ethephon concentration and wet weight was not linear, and differences between ethephon treatments were not significant. Ethephon did not influence the thickness of *K. xylinus* pellicles (data not shown).

Similar to the wet weights, pellicle dry weight was affected by ethephon. All concentrations of ethephon resulted in a significant increase in pellicle dry weight (**Figure 4B**). Dry weights increased linearly with ethephon concentration in a dose-dependent manner, although differences between ethephon treatments were not statistically significant. In comparison to the untreated control, a 23% increase in BC yield was observed when *K. xylinus* was grown in the presence of 0.01 mM ethephon, while a 27 and 29% increase was observed after ethephon treatment at concentrations of 0.1 and 1.0 mM, respectively.

Ethephon significantly decreased pellicle hydration compared to the untreated control (**Figure 4C**). We hypothesized that the decrease in pellicle hydration was related to differences in crystallinity. To test this, the crystallinity index, CI(IR), of *K. xylinus* pellicles produced in the presence of ethephon was determined. Compared to the untreated control, pellicles grown in the presence of all concentrations of ethephon had a higher crystallinity (**Figure 4D**). The CI(IR) of untreated pellicles was 0.62, while pellicles grown with ethephon treatments of 0.01, 0.1, and 1.0 mM had a significantly increased CI(IR) of 0.69, 0.68, and 0.73, respectively. Therefore pellicle crystallinity increased by 11, 10, and 16% when synthesized in the presence of 0.01, 0.1, and 1.0 mM ethephon, respectively (**Figure 4D**). This is consistent with the observed decrease in pellicle hydration after ethephon treatment. In the presence of ethephon, the pellicles were more crystalline and therefore less able to retain water. Control experiments with chloride and phosphate yielded results comparable to untreated cultures, indicating the observed phenotypes were caused by ethylene.

### Ethylene Increases *K. xylinus* BC Production on Solid Medium

**FIGURE 5:**
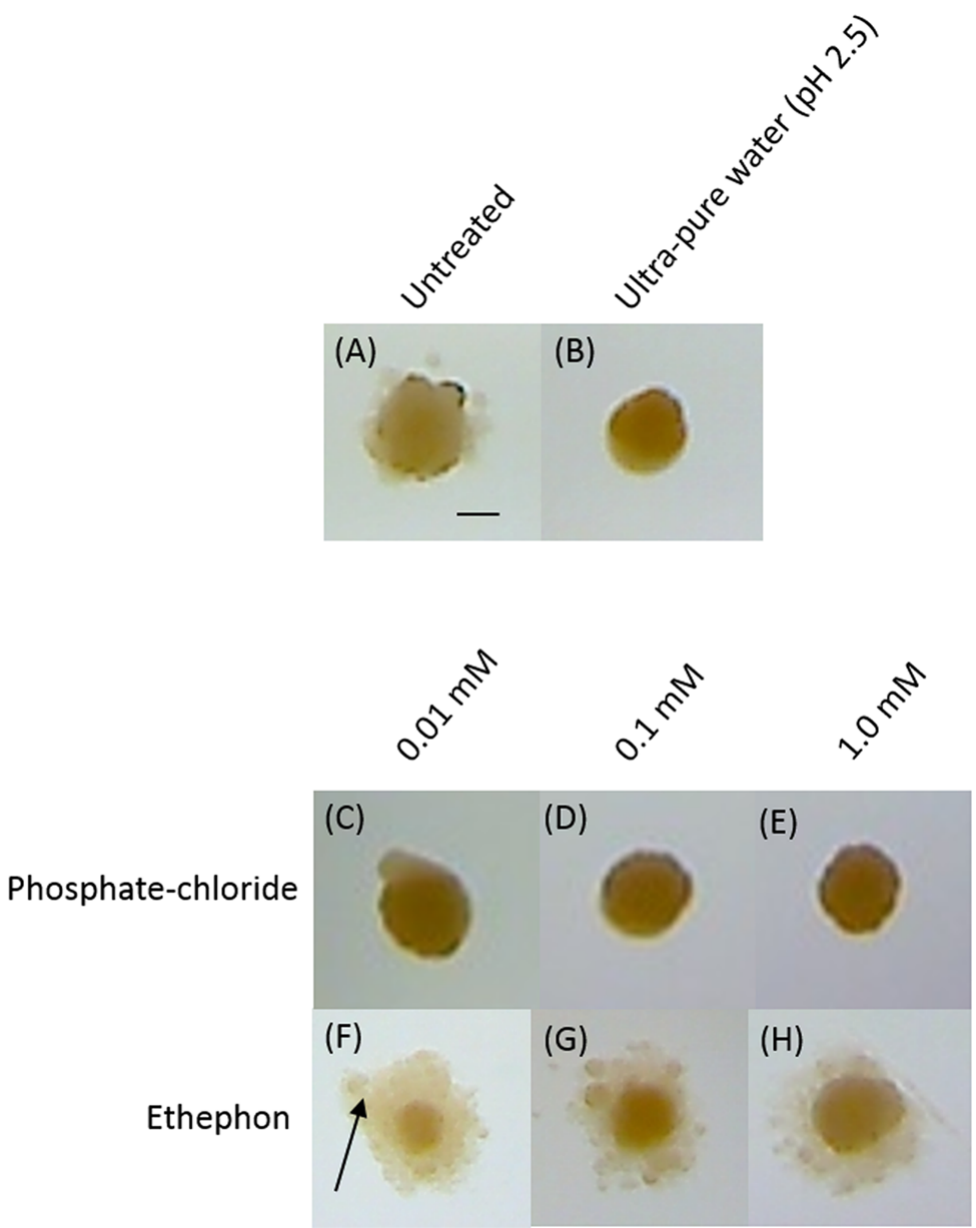
**Ethephon enhances *K. xylinus* BC production when grown on solid medium.** *K. xylinus* was streaked onto SH agar plates (pH 7) that were untreated **(A)**, or pre-treated with ultra-pure water (pH 2.5); **(B)** phosphate and chloride **(C-E)** or ethephon **(F-H)**. Plates were incubated at 30°C for 7 days. Representative colonies are shown. The arrow points to BC, seen as the hazy substance surrounding the colony. Scale bar represents 0.5 mm.

In order to assess how ethylene affected *K. xylinus* colony morphology and BC production on solid medium, agar plates were pre-treated with ethephon, acidified ultra-pure water (pH 2.5), or phosphate-chloride, then streaked for isolated colonies. **Figure 5** shows representative colonies from the various treatments. Colonies formed under control conditions (untreated) were about 1 mm in diameter, convex in elevation, irregular in form, and slightly orange in color (**Figure 5A**). All treatments, including the acidified water (**Figure 5B**) and phosphate-chloride (**Figures 5C-E**) controls influenced BC production of agar-grown cultures. Colonies grown on untreated plates produced some BC, as shown by the hazy material surrounding the central part of the colony (**Figure 5A**). Interestingly, when plates were pre-treated with acidified ultra-pure water (**Figure 5B**) or phosphate-chloride (**Figures 5C-E**), *K. xylinus* colonies produced minimal BC. Colonies grown in the presence of ethephon (**Figures 5F-H**) produced more BC than all of the controls, with the largest increase observed for the 0.01 mM ethephon treatment (**Figure 5F**). Colonies grown on the phosphate-chloride control plates were similar to those grown with acidified ultra-pure water, demonstrating that the ethephon-induced BC over-production phenotype was caused by ethylene. This result is reinforced by the increased BC yield observed for statically grown liquid cultures exposed to ethylene (**Figure 4B**).

### Ethylene and IAA Cause Differential Expression of *bcs* Operon Genes

**FIGURE 6:**
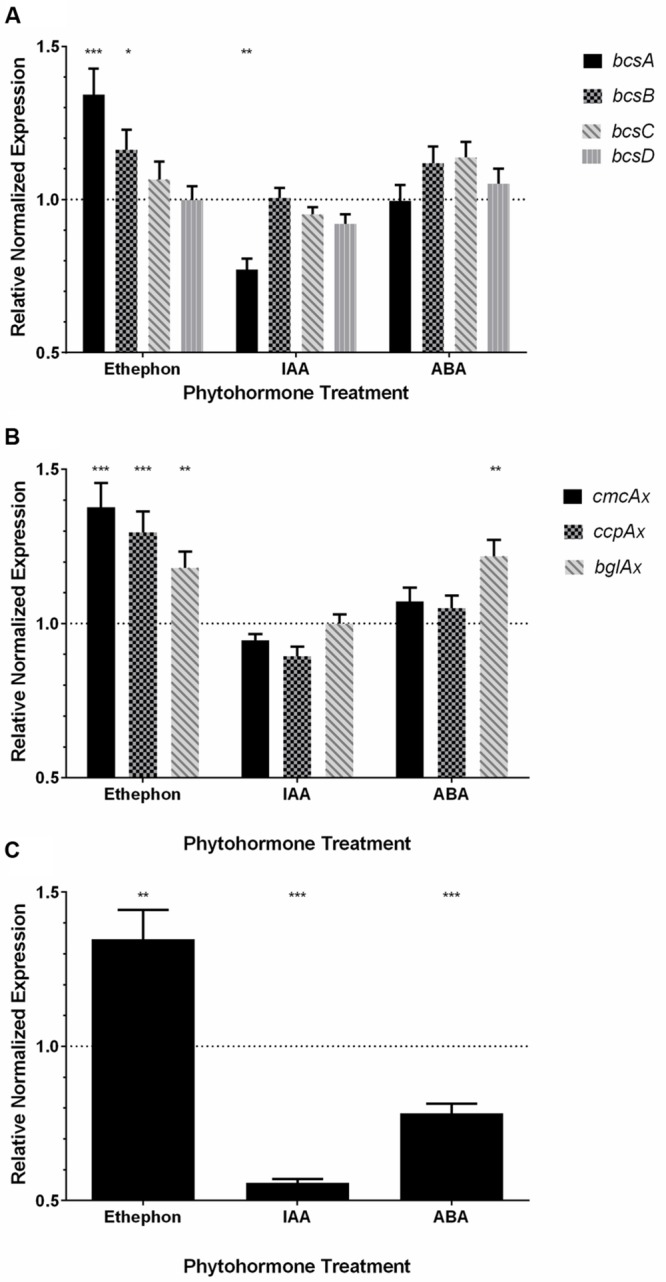
**The expression of genes involved in *K. xylinus* BC biosynthesis are regulated by phytohormones.** Ethephon-derived ethylene and IAA induce differential expression of the genes within the *bcs* operon **(A)**. Ethephon-derived ethylene and ABA influence the expression of genes flanking the *K. xylinus bcs* operon **(B)**. Ethephon-derived ethylene, IAA and ABA regulate the expression of *crp/fnr_Kx_* **(C)**. Gene expression was quantified using RT-qPCR after treatment with 10 μM ethephon, 10 μM IAA, or 10 μM ABA. Expression values were made relative to the respective untreated controls and normalized using the expression values of reference genes, *23SrRNA* and *gyrB*. The dotted line indicates the relative normalized expression value for the untreated control. Error bars show the SD (*n* = 3). ^∗^*p* < 0.05; ^∗∗^*p* < 0.01; ^∗∗∗^*p* < 0.001.

All primer sets produced amplification efficiencies between 90–110%, as shown by the standard curves (Supplemental Figures S4–S13; Supplemental Table S2), indicating all assays were robust. The expression of the *bcs* operon genes (**Figure 1B**), *bcsA, bcsB, bcsC, and bcsD*, encoding proteins that form the BC synthase complex responsible for BC synthesis (**Table 1**; **Figure 1A**), were analyzed from *K. xylinus* cultures grown in the presence of 10 μM ethephon, 10 μM IAA, and 10 μM ABA. When cultures were treated with ethephon, *bcsA*, and *bcsB* were up-regulated by 34% (*p* < 0.001) and 16% (*p* < 0.05), respectively, compared to the untreated control (**Figure 6A**). Interestingly, *bcsC* and *bcsD* were not affected. The phosphate-chloride control treatment caused no differences in the expression of the *bcs* operon genes (data not shown), supporting the conclusion that the observed differential expression of these genes was caused by ethephon-derived ethylene. IAA treatment down-regulated *bcsA* by 23% (*p* < 0.01) compared to the untreated control, while *bcsB, bcsC*, and *bcsD* were not affected (**Figure 6A**). The expression of all *bcs* operon genes were unaffected by ABA treatment (**Figure 6A**). Ethylene and IAA therefore uniquely cause differential expression of the genes within the *K. xylinus bcs* operon. Ethylene up-regulates *bcsA* and *bcsB* which is consistent with the increase in BC yield (**Figures 4B** and **5F-H**). IAA down regulates *bcsA* while ABA has no effect on the expression of *bcs* genes which is consistent with our previous observation that IAA directly decreases BC production while ABA had an indirect effect on BC yield in *K. xylinus* (Qureshi et al., 2013).

### Ethylene and ABA Affect the Expression of Genes Flanking the *bcs* Operon

Three other genes known to be involved in *K. xylinus* BC biosynthesis were also analyzed via RT-qPCR. The *ccpAx* and *cmcAx* genes form an operon upstream, while *bglAx* is located downstream of the *bcs* operon (**Figure 1B**; **Table 1**). Ethephon-derived ethylene caused significant up-regulation of *ccpAx* (30%, *p* < 0.001), *cmcAx* (38%, *p* < 0.001) and *bglAx* (18%, *p* < 0.01) compared to the untreated control (**Figure 6B**). The expression of *bglAx* was significantly up-regulated 22% (*p* < 0.01) compared to the control after treatment with ABA, while IAA treatment had no effect on these genes (**Figure 6B**).

### The CRP/FNR_Kx_ Transcription Factor Gene is Hormonally Regulated

The expression of the *crp/fnr_Kx_* gene (H845_3156), bioinformatically identified in the genome of *K. xylinus* E25, was assessed by RT-qPCR to determine if it is regulated by ethylene, IAA, or ABA. This gene was studied since it has high similarity to a *crp/fnr_Kh_* gene in *K. hansenii* ATCC 23769 (GXY_00863) that has recently been shown to be essential for BC biosynthesis (Deng et al., 2013). RT-qPCR primers were designed based on the nucleotide sequence of a *crp/fnr_Kx_* gene (H845_3156) from the *K. xylinus* E25 genome. Primers were validated using *K. xylinus* ATCC 53582 gDNA as the template (data not shown). The expression of *crp/fnr_Kx_* is regulated by ethylene, IAA, and ABA (**Figure 6C**). Compared to the untreated controls, ethephon-derived ethylene significantly up-regulated *crp/fnr_Kx_* by 35% (*p* < 0.01), while IAA and ABA down-regulated its expression by 45% (*p* < 0.001) and 22% (*p* < 0.001), respectively. Taken together, these results indicate that *crp/fnr_Kx_* is phytohormonally regulated. Ethylene directly increases BC production (**Figures 4B** and **5F-H**) and up-regulates *crp/fnr_Kx_* expression (**Figure 6C**), while IAA directly decreases BC production (Qureshi et al., 2013) and down-regulates *crp/fnr_Kx_* expression (**Figure 6C**). This suggests that like CRP/FNR_Kh_ in *K. hansenii* (Deng et al., 2013), CRP/FNR_Kx_ may directly regulate BC biosynthesis in *K. xylinus*.

## Discussion

Ethephon, an ethylene-releasing compound, was used to investigate the effect of ethylene on *K. xylinus* ATCC 53582 growth, BC production, pellicle properties and gene expression. *K. xylinus* is a plant-associated carposphere bacterium (Park et al., 2003; Dellaglio et al., 2005; Jahan et al., 2012; Neera et al., 2015). This close association with fruit exposes *K. xylinus* to a plethora of plant-derived compounds, including phytohormones that regulate plant growth and development when present at low concentrations (Davies, 2010). Ethylene is the main ripening hormone in climacteric fruit and is released in high concentrations during the ripening stage (Pech et al., 2008; McAtee et al., 2013). In non-climacteric fruit, ABA is believed to be the most important ripening hormone (Jia et al., 2011; Li et al., 2011; McAtee et al., 2013). However, recent data has shown that even non-climacteric fruit, such as strawberry, grapes, and citrus respond to ethylene and experience a spike in ethylene production during ripening, though the magnitude of response and production is cultivar-dependent (Chervin et al., 2004; Trainotti et al., 2005; Iannetta et al., 2006; Paul et al., 2012). Nevertheless, *K. xylinus* would be exposed to exogenous ethylene when it inhabits both climacteric and non-climacteric fruit in the environment.

The ripening stage of fruit development is characterized by modulation of endogenous hormone levels (McAtee et al., 2013). Numerous metabolic changes occur that result in the fruit being sweeter, due to starch hydrolysis by amylase (Agravante et al., 1990) and softer, due to the activity of pectinase and cellulase enzymes that degrade the fruit cell wall (Ahmed and Labavitch, 1980; Sitrit and Bennett, 1998; Lohani et al., 2004). Due to the intense turgor pressure in plant cells, weakening of the cell wall results in the release of exudate onto the fruit surface (Brummell and Harpster, 2001; Brummell, 2006), providing an attractive nutrient environment for microorganisms. The levels of ethylene are typically low during fruit growth and maturation, but spike at the onset of ripening (McAtee et al., 2013). Ethylene, due to its positive role in fruit ripening, would therefore be a signal indicating an ideal nutrient environment for *K. xylinus* as fruit at this developmental stage contain high levels of soluble monosaccharides that can be used for growth and BC production.

Investigations using ethephon in plants have shown ethylene-dependent phenotypes even though the dosage and magnitude of ethephon decomposition was not precisely controlled (Zhang and Wen, 2010; Zhang et al., 2010). In this study, ethephon was used as a convenient vehicle to produce *in situ* ethylene when added to *K. xylinus* cultures growing in SH medium. Ethephon decomposition is linear, in that the amount of ethylene produced is proportional to the amount of ethephon used (Klein et al., 1979; Zhang and Wen, 2010). The half-life in aqueous medium at 30°C is 5.1 ± 0.9 h at pH 7 and 6.0 ± 0.9 h at pH 6 (Klein et al., 1979). Biddle et al. (1976) demonstrated that ethephon decomposition below pH 5 is extremely slow, while the rate is greatly increased at pH 7. The *A. thaliana* triple response assay verified that ethephon decomposition and ethylene production occurred in SH medium (pH 7) and time-course pH analysis showed that the decrease in culture pH would not have hampered this process. Ethephon decomposition results in the production of chloride and phosphate ions in addition to the desired ethylene. Therefore, control experiments using chloride and phosphate were completed alongside the ethephon experiments. In all cases, the effects of phosphate and chloride were insignificant, supporting our conclusion that the observed results were due to ethylene.

Minimum inhibitory concentration assays were performed to ensure ethephon was not toxic to *K. xylinus*. Ethephon concentrations of 50 and 100 mM prevented *K. xylinus* growth. Control assays revealed that the bacterium could grow when chloride and phosphate concentrations were at 50 mM but not 100 mM, suggesting that ethylene was responsible for *K. xylinus* growth inhibition when the medium was supplemented with 50 mM ethephon. To our knowledge, this is the first time ethephon has been used to treat bacteria with ethylene. However, there are numerous literature reports that describe treating plants with millimolar concentrations of ethephon (Denney and Martin, 1994; Khan, 2004; Kong et al., 2009; Dhillon and Mahajan, 2011; Wang et al., 2013). Burg and Burg (1962) showed that ethylene production levels in fruit vary depending on the cultivar. The internal ethylene concentration of various fruit varieties is in the micromolar range, with the exception of apples and passion fruit, which produce ethylene in the high mircomolar to low millimolar range. Furthermore, Wheeler et al. (2004) demonstrated that the peak ethylene concentration in ripening tomatoes is approximately 10 μM. Based on this, an ethylene concentration of 50 mM is not physiologically relevant, and therefore the non-toxic ethephon-derived ethylene concentrations of 0.01, 0.1, and 1.0 mM were utilized in our study.

Ethylene reduced pellicle hydration by increasing pellicle crystallinity, suggesting a higher degree of hydrogen bonding between adjacent glucan chains that resulted in a reduced ability to hydrogen bond to water. Qureshi et al. (2013) showed that exogenous IAA, ABA, GA_3_, and zeatin influenced the hydration and crystallinity of *K. xylinus* pellicles in a concentration-dependant manner. In contrast, all concentrations of ethylene decreased hydration and increased the crystallinty of the pellicles in this study. Increased crystallinity enhances the recalcitrance of BC, which provides a survival advantage to *K. xylinus* in the environment since it would be less susceptible to cellulase-producing microorganisms.

An increase in BC yield by *K. xylinus* is a direct result of exposure to ethylene in both liquid and solid media; growth is not affected. We observed an increase in BC production after ethylene treatment, further supporting the conclusion that it has a direct effect on BC biosynthesis. This is in contrast to ABA, zeatin, and GA_3_ which indirectly enhance BC production due to an increased growth rate (Qureshi et al., 2013). Hu and Catchmark (2010) investigated the effect of 1-methylcyclopropene (1-MCP), a known inhibitor of ethylene-dependant responses in plants (Tassoni et al., 2006), on the growth and BC production of *K. xylinus*. This compound is thought to antagonistically bind ethylene receptors and blocks the effects of ethylene in plants (Sisler, 1991). Though an ethylene receptor has not been reported for *K. xylinus*, Hu and Catchmark (2010) showed that 1-MCP decreased *K. xylinus* growth rate and increased BC yield, indicating that similar to ethylene, 1-MCP has a direct effect on BC biosynthesis. This suggests that an ethylene receptor exists in *K. xylinus* and that ethylene and 1-MCP bind the same receptor since they induce similar phenotypes. This is in contrast to their effects on fruit, since treatment with 1-MCP inhibits ethylene-dependent ripening phenotypes (Tassoni et al., 2006). The direct target of ethylene and 1-MCP in *K. xylinus* has not yet been identified.

The analysis of pH changes over the course of *K. xylinus* growth revealed that the final culture pH is higher after treatment with ethylene. We observed a decrease in pH during exponential growth and then an increase in pH during stationary growth phase. This was also observed by Kawano et al. (2002b), who correlated this trend in pH alteration to glucose oxidation into gluconic acid, and subsequent resorption of gluconic acid. They also showed that unlike *K. hansenii* ATCC 23769, *K. xylinus* ATCC 53582 is able to synthesize BC after glucose depletion by using gluconic acid as carbon source. The higher final pH and increased production of BC observed in this study may therefore be attributed in part to increased gluconic acid metabolism in the presence of ethylene. However, this requires further investigation.

We report for the first time, the differential expression of genes within the *K. xylinus bcs* operon. This operon encodes proteins that comprise the complex responsible for BC production in *K. xylinus*. The *bcs* operon in *K. xylinus* encodes four genes (*bcsABCD*) which are believed to form a polycistronic mRNA. However, little is known about how the *bcs* operon is regulated, or how relative gene expression is affected by external signals. Ethylene up-regulated the expression of *bcsA* and *bcsB*, which encode enzymes responsible for BC biosynthesis, but not *bcsC* and *bcsD*, which encode enzymes for BC export and crystallization. Therefore, an increase in BC production only requires additional synthesis proteins (BcsA and BcsB), but not increased levels of export/crystallization proteins (BcsC and BcsD). Similarly, IAA down-regulated *bcsA* but did not affect the other *bcs* genes. Since *bcsA* encodes the BC synthase (BcsA) that is responsible for synthesis of BC, it follows that its down-regulation results in decreased BC production as observed with IAA, and that its up-regulation results in increased BC production as observed with ethylene. This data indicates that ethylene and IAA directly influence BC biosynthesis at a transcriptional level. In order to increase BC biosynthesis, both *bcsA* and *bcsB* must be up-regulated, since functional BcsA is stabilized by BcsB in the periplasm (Morgan et al., 2013). However, decreasing BC biosynthesis only requires the down-regulation of *bcsA*, since its protein product directly synthesizes BC. It is possible that *K. xylinus* preserves cellular energy by maintaining constitutive levels of *bcsB* transcription so that the transcript is readily available for translation into BcsB once *bcsA* repression is relieved.

The differential relative expression of genes within operons is especially useful for regulating encoded proteins that serve different functions, such as the protein products of the *bcs* operon genes. BcsA synthesizes BC, BcsB chaperones BC chains through the periplasm, BcsC facilitates export of BC into the extracellular environment and BcsD is responsible for crystallization of BC. In some cases, differential relative expression of genes within an operon can be attributed to different translation efficiencies of operon-encoded mRNA due to variations in ribosome-binding sites (Vellanoweth, 1993). Inhibition of individual genes in a polycistronic mRNA by anti-sense RNA causes differential relative gene expression of the *E. coli* galactose operon (Møller et al., 2002) and various other operons, including those regulated by excludons (Sesto et al., 2013; Lasa and Villanueva, 2014). Lastly, polycistronic mRNA can undergo RNase cleavage and produce individual mRNAs that differ in stability, leading to differential protein expression. For example, the genes within the *Escherichia coli atp* operon that encode proteins that make up the ATP synthase (Gay and Walker, 1981) are differentially regulated (McCarthy et al., 1991). Like the BC synthesis complex, the ATP synthase is made up of numerous protein subunits and is embedded in the cell membrane of Gram-negative bacteria. The differential expression of the *atpIBEFHAGDC* operon genes has been attributed to segmental differences in mRNA stability (McCarthy et al., 1991; Lagoni et al., 1993); the mRNA of the first two genes of the operon are rapidly degraded by RNase enzymes (Ziemke and McCarthy, 1992), while the remaining seven genes are more stable (McCarthy et al., 1991). In addition, the translational efficiencies of mRNA from the *atp* genes vary (Brusilow et al., 1982) so that the differential expression of the operon is controlled at two post-transcriptional levels. This type of regulation has also been observed for the *malEFG* operon in *E. coli* (Newbury et al., 1987), the *gap-pgk* operon of *Zymomonas mobilis* (Eddy et al., 1991; Burchhardt et al., 1993) and *Clostridium acetobutylicum* (Schreiber and Dürre, 2000), the *dnaK* operon of *B. subtilis* (Homuth et al., 1999), the *puf* operon in *Rhodobacter capsulatus* (Belasco et al., 1985; Klug et al., 1987) and the uropathogenic *E. coli pap* operon (Båga et al., 1988; Nilsson et al., 1996). It is therefore possible that the *bcs* operon in *K. xylinus* is differentially regulated through one of these mechanisms.

The deletion of *ccpAx* in *K. hansenii* ATCC 23769 significantly decreased levels of BcsB and BcsC, but not BcsA, which are controlled by the same promoter (Deng et al., 2013). The expression of *bcsD* is regulated by a different promoter (Deng et al., 2013) supporting the notion that differential expression of the genes that make up the BC synthesis complex does occur. Therefore, it is possible that in *K. hansenii, ccpAx* plays a role in regulating differential relative expression of the *bcsABC* operon leading to the differential protein expression observed in the study by Deng et al. (2013).

**FIGURE 7:**
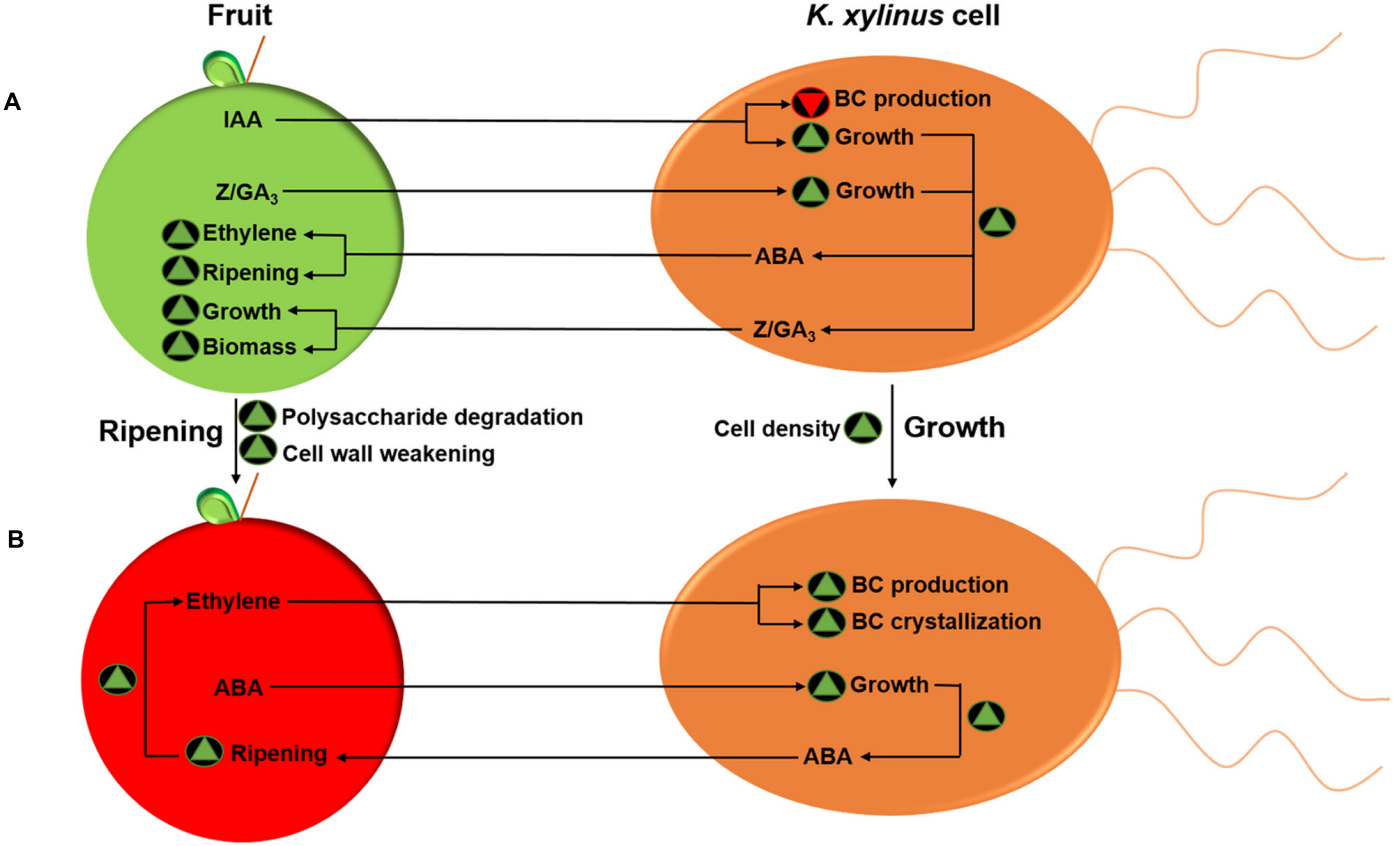
**Model for the phytohormone-mediated fruit-bacteria interactions of *K. xylinus*.** Unripe fruit **(A)** contain high concentrations of IAA, zeatin (Z) and GA_3_. IAA decreases *K. xylinus* BC production, while IAA, zeatin, and GA_3_ increase bacterial cell growth enhancing endogenous production of ABA, zeatin, and GA_3_ by *K. xylinus*. These hormones increase fruit size and induce ripening, characterized by degradation of polysaccharides and weakening of the fruit cell wall. On ripe fruit **(B)**, plant-produced ethylene up-regulates biosynthesis and crystallization of BC. Exogenous ABA increases *K. xylinus* growth, allowing it to accelerate the fruit ripening process, which facilitates colonization. Green triangles and inverted red triangles indicate a process is enhanced or repressed, respectively. Only direct effects are shown.

Based on the results of the current study, as well as data provided by Qureshi et al. (2013), we propose a model for the phytohormone-mediated fruit-bacteria interactions of *K. xylinus* (**Figure 7**). On unripe fruit (**Figure 7A**), *K. xylinus* is exposed to high concentrations of IAA, zeatin (Z), and GA_3_. As a fruit ripening inhibitor (Frenkel and Dyck, 1973; Davies et al., 1997; Symons et al., 2012; Ziliotto et al., 2012), IAA directly inhibits energetically costly BC biosynthesis by down-regulating *bcsA*, since carbon source is limited on unripe fruit. Exogenous IAA, zeatin and GA_3_ increase *K. xylinus* growth, enhancing bacterial production of zeatin and GA_3_. These two hormones increase fruit size by controlling cytokinesis (zeatin) and cell enlargement (GA_3_; McAtee et al., 2013). Previous studies showed that rhizosphere bacteria that produce endogenous zeatin and GA_3_ can enhance plant growth (Bottini et al., 2004; Arkhipova et al., 2005) and that application of GA_3_ increases the size of grape berries (Casanova et al., 2009) and tomatoes (Serrani et al., 2007). Therefore, we postulate that endogenous production of zeatin and GA_3_ by *K. xylinus* may contribute to the pool of endogenous hormone levels in the fruit so that there is more biomass to colonize once ripening begins. Endogenous production of ABA is also increased as a result of enhanced cell growth. Exposing fruit to exogenous ABA increases endogenous ABA levels in fruit tissues, triggers ethylene biosynthesis and induces ripening (Zhang et al., 2009; McAtee et al., 2013; Leng et al., 2014). ABA production by *K. xylinus* may therefore play a role in triggering ripening, resulting in a preferred growth environment compared to unripe fruit. Furthermore, IAA-, zeatin-, and GA_3_-mediated growth enhancement ensures that cell density is at its peak when ripening begins. On ripe fruit (**Figure 7B**), *K. xylinus* would be exposed to high concentrations of ABA and ethylene (McAtee et al., 2013). Fruit-produced ABA increases *K. xylinus* growth (Qureshi et al., 2013), increasing bacterial production of ABA, which in turn up-regulates plant-produced ethylene and accelerates ripening (Zhang et al., 2009). Our hypothesis that ABA does not directly influence BC biosynthesis is supported by the observation that it did not affect the expression of genes within the *bcs* operon. In contrast, exogenous ethylene directly enhances BC biosynthesis in *K. xylinus* by up-regulating the expression of *bcsA* and *bcsB*. Therefore, ABA and ethylene act together as environmental signals to promote colonization of ripe fruit by *K. xylinus* through increased cell growth and direct enhancement of BC biosynthesis. Over production of BC provides a competitive advantage to *K. xylinus* as shown by apple colonization studies (Williams and Cannon, 1989). Enhancement of BC production and crystallization by ethylene therefore increases the environmental fitness of *K. xylinus* within the carposphere.

Ethylene also up-regulated *cmcAx* and *ccpAx* which form their own operon upstream of the *bcs* operon (Kawano et al., 2002b; Sunagawa et al., 2013). CcpAx is essential for BC biosynthesis (Standal et al., 1994) and is believed to be involved in crystallizing BC due to its localization with BcsD (Sunagawa et al., 2013). The precise function and transcriptional regulation of CcpAx is not known. Similarly, the exact function of CmcAx is not known. However, it is required for BC biosynthesis as CmcAx inhibition by antibodies results in decreased BC production (Koo et al., 1998) and addition of minute amounts of CmcAx, or its overexpression increases BC production (Tonouchi et al., 1995; Kawano et al., 2002a, 2008). CmcAx is believed to play a role in editing distorted glucan chains since *cmcAx* deletion results in the formation of highly twisted ribbons (Nakai et al., 2013). Expression of *cmcAx* is induced by gentiobiose produced by BglAx (Kawano et al., 2008). The expression of *bglAx* is induced by the CRP/FNR_Kh_ transcription factor in *K. hansenii* ATCC 23769 (Deng et al., 2013). We demonstrated that ethylene and ABA up-regulate the expression of *bglAx*. The ethylene-dependant up-regulation of all three genes may be required to cope with the enhancement of BC biosynthesis. In addition, up-regulation of *cmcAx* and *bglAx* may lead to increased levels of secreted CmcAx and BglAx which have cellulose-hydrolyzing activity (Tahara et al., 1998; Tajima et al., 2001; Kawano et al., 2002b). These proteins may aid in the degradation of plant cellulose in order to weaken the fruit cell wall and provide more glucose for BC production.

The CRP/FNR family of transcription factors regulate the expression of genes critical to bacterial growth and survival in response to changing environmental conditions (Körner et al., 2003). Through a bioinformatics approach, we identified a novel phytohormone-regulated CRP/FNR transcription factor (CRP/FNR_Kx_) in *K. xylinus* ATCC 53582. Ethylene up-regulated *crp*/*fnr_Kx_* expression and increased BC production. IAA down-regulated *crp*/*fnr_Kx_* expression and decreased BC production. Together, these results suggest that CRP/FNR_Kx_ directly regulates BC biosynthesis in *K. xylinus* at a transcriptional level, similar to how CRP/FNR_Kh_ regulates BC biosynthesis in *K. hansenii* (Deng et al., 2013). Positive regulation of biofilm formation by CRP/FNR family protein, CRP, has also been observed in *E. coli* (Jackson et al., 2002) and *Shewanella oneidensis* (Thormann et al., 2005) when bound to cAMP. In contrast, CRP-cAMP negatively regulates transcription of genes involved in the biosynthesis of *Vibrio* polysaccharide (VPS) in *Vibrio cholerae* (Fong and Yildiz, 2008). While CRP/FNR_Kx_ positively regulates BC production in *K. xylinus*, ABA down-regulates *crp/fnr_Kx_* but does not decrease BC yield (Qureshi et al., 2013). Since this study focused on a small subset of genes, it is likely that ABA regulates a yet to be identified gene whose protein product counteracts the down-regulation of *crp/fnr_kx_* underscoring the complexity of the regulatory pathway involved in controlling BC synthesis.

The CRP/FNR family transcription factor, Bcam1349, binds c-di-GMP and regulates biofilm formation by enhancing the production of BC and curli fimbriae in *Burkholderia cenocepacia* (Fazli et al., 2011). Binding of c-di-GMP enhanced the ability of Bcam1349 to bind the promoter region and increase the expression of *bcs* operon genes (Fazli et al., 2011) along with the *Bcam1330-Bcam1341* gene cluster involved in exopolysaccharide production (Fazli et al., 2013). Interestingly, various diguanylate cyclases and phosphodiesterases involved with c-di-GMP metabolism are regulated by the CRP/FNR family protein, CRP, in *V. cholerae* (Fong and Yildiz, 2008). Whether CRP/FNR_Kx_ binds c-di-GMP, or regulates the expression of diguanylate cyclases and phosphodiesterases remains to be investigated.

CRP/FNR_Kh_ regulates BC biosynthesis in *K. hansenii* ATCC 23769 by positively regulating the expression of *bglAx* and other genes required for BC production that have yet to be identified (Deng et al., 2013). Ethylene up-regulates both *crp*/*fnr_Kx_* and *bglAx*. CRP/FNR_Kx_ may positively regulate *bglAx* transcription in *K. xylinus*. In contrast, ABA induces inverse expression of *crp*/*fnr_Kx_* and *bglAx*, while IAA down-regulates *crp*/*fnr_Kx_*, but has no effect on *bglAx* suggesting that the mechanisms involved in CRP/FNR_Kx_-mediated transcriptional regulation are influenced differently by ethylene, IAA and ABA. This may be explained by the complex regulatory role that CRP/FNR family transcription factors have in bacteria, due to their ability to regulate numerous target genes depending on activation by a ligand. For example, the *E. coli* CRP transcription factor activates the transcription of over 100 promoters (Kolb et al., 1993; Harman, 2001; Brown and Callan, 2004). The activation of CRP is dependent on allosteric binding of cAMP (Harman, 2001). Apo-CRP has low DNA binding affinity and displays minimal sequence specificity. Binding of cAMP results in conformational changes that result in high affinity, sequence-specific DNA binding and interaction with RNA polymerase (Won et al., 2009). Sigma factors produced under the specific conditions then coordinate sequence-specific gene expression mediated by active CRP and RNA polymerase (Colland et al., 2000). Therefore, it is possible that ethylene, IAA and ABA alter the DNA binding specificity of CRP/FNR_Kx_ resulting in differential regulation of *bglAx*. To our knowledge, this is the first report of a bacterial CRP/FNR family transcription factor that is regulated by phytohormones.

## Conclusion

We have demonstrated that ethylene produced through the *in situ* decomposition of ethephon can be used to study the effects of this hormone on bacteria. To our knowledge, we are the first group to utilize this approach. Ethylene caused an increase in *K. xylinus* BC biosynthesis directly by up-regulating the expression of *bcsA* and *bcsB* and indirectly by up-regulating the expression of *cmcAx, ccpAx* and *bglAx*. IAA decreases BC biosynthesis directly by down-regulating *bcsA* expression. We also showed that the *bcs* operon in *K. xylinus* is differentially regulated by ethylene and IAA. Altogether, we have expanded on the putative fruit-bacteria interactions of *K. xylinus*, provided new insights into the transcriptional regulation of the *bcs* operon and identified a new phytohormone-regulated CRP/FNR_Kx_ transcription factor that plays a role in BC biosynthesis in *K. xylinus* ATCC 53582.

## Author Contributions

JS conceived the research topic, contributed to the experimental design, and co-wrote the manuscript. RA carried out the experimental work, contributed to the experimental design, and co-wrote the manuscript.

## Conflict of Interest Statement

The authors declare that the research was conducted in the absence of any commercial or financial relationships that could be construed as a potential conflict of interest.

## Footnotes

1 http://www.ncbi.nlm.nih.gov/tools/primer-blast/

2 http://mfold.rna.albany.edu/?q=mfold/dna-folding-form
