# Supplementary material for: The Phytohormone Ethylene Enhances Cellulose Production, Regulates CRP/FNRKx Transcription and Causes Differential Gene Expression within the Bacterial Cellulose Synthesis Operon of Komagataeibacter (Gluconacetobacter) xylinus ATCC 53582
Source: Front Microbiol. 2015 Dec 22;6:1459. doi: 10.3389/fmicb.2015.01459 (PMC4686702; doi:10.3389/fmicb.2015.01459)
Supplement: Supplementary file 1 [file Presentation_1.PPTX]

## Slide 1
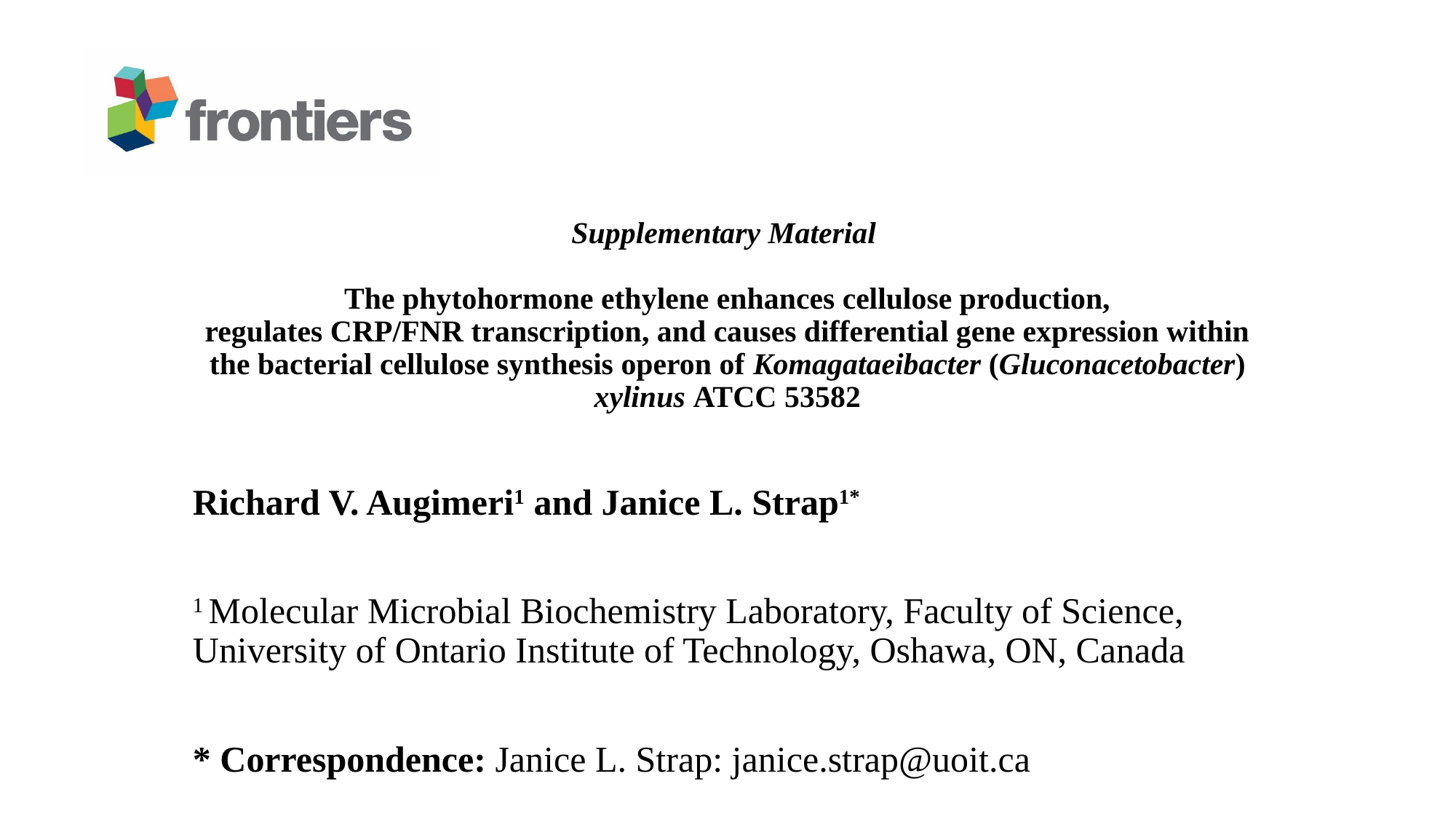

# Supplementary Material The phytohormone ethylene enhances cellulose production, regulates CRP/FNR transcription, and causes differential gene expression within the bacterial cellulose synthesis operon of Komagataeibacter (Gluconacetobacter) xylinus ATCC 53582
Richard V. Augimeri1 and Janice L. Strap1*
1 Molecular Microbial Biochemistry Laboratory, Faculty of Science, University of Ontario Institute of Technology, Oshawa, ON, Canada
* Correspondence: Janice L. Strap: janice.strap@uoit.ca

## Slide 2
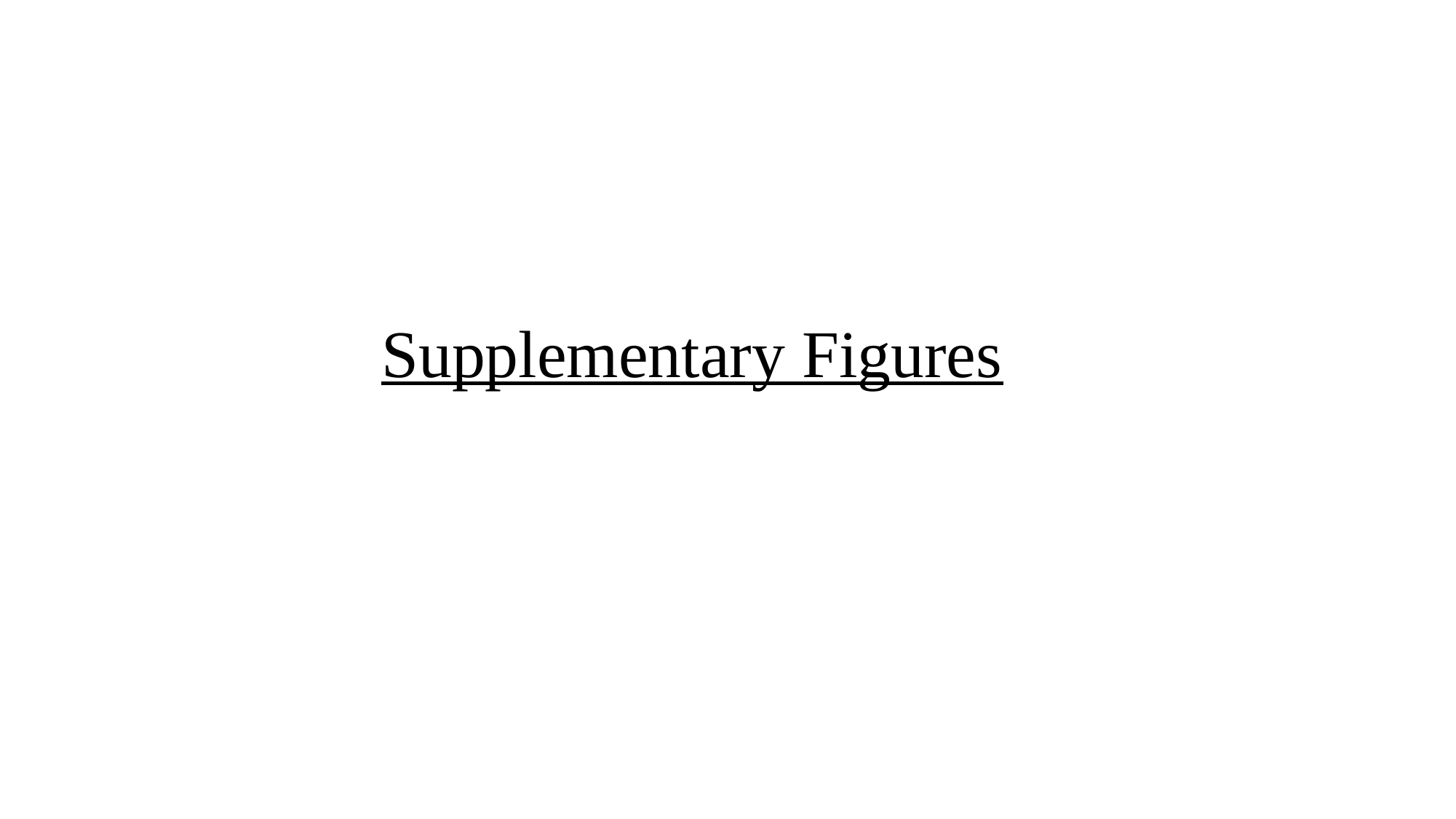

# Supplementary Figures

## Slide 3
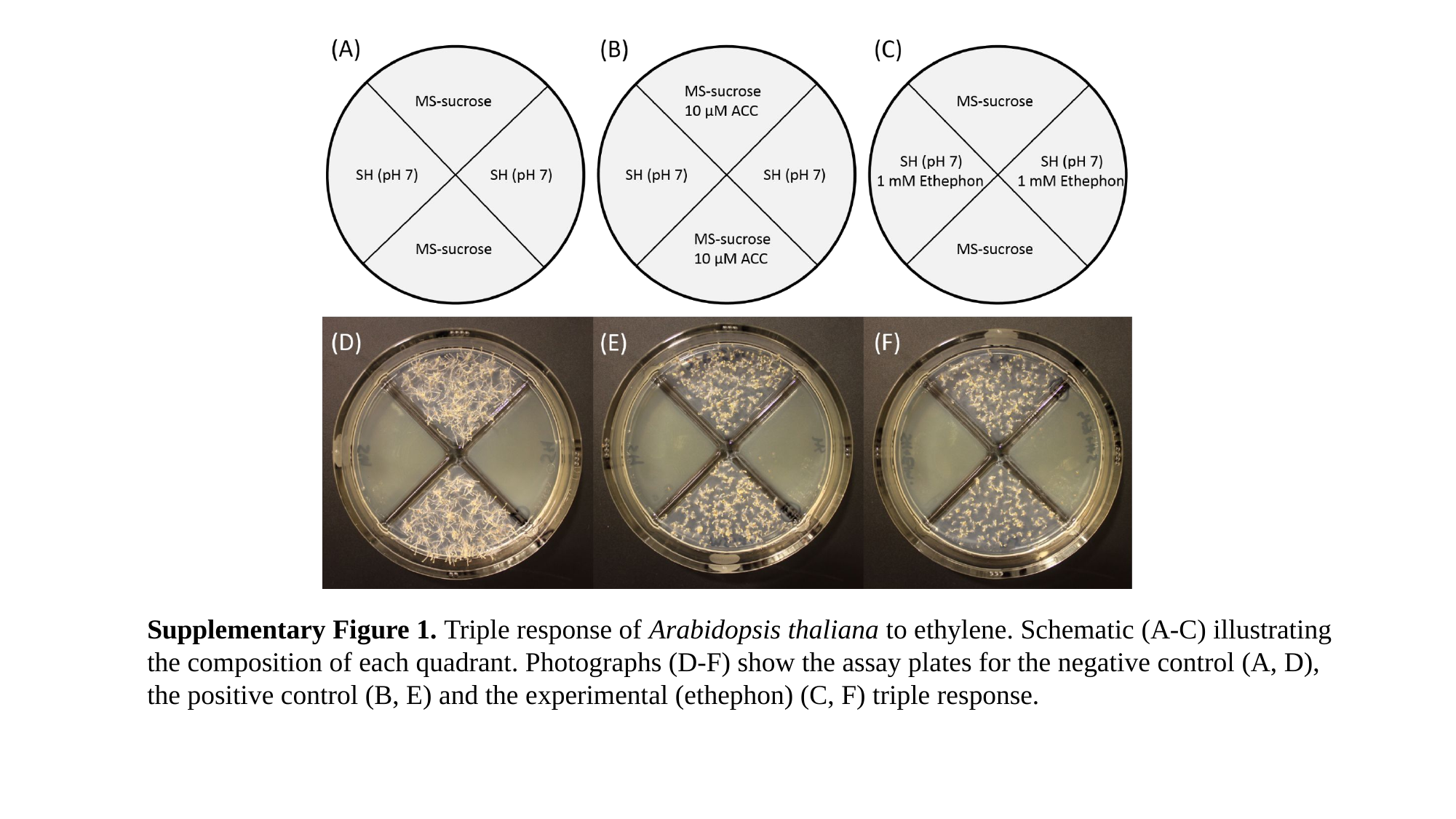

Supplementary Figure 1. Triple response of Arabidopsis thaliana to ethylene. Schematic (A-C) illustrating the composition of each quadrant. Photographs (D-F) show the assay plates for the negative control (A, D), the positive control (B, E) and the experimental (ethephon) (C, F) triple response.

## Slide 4
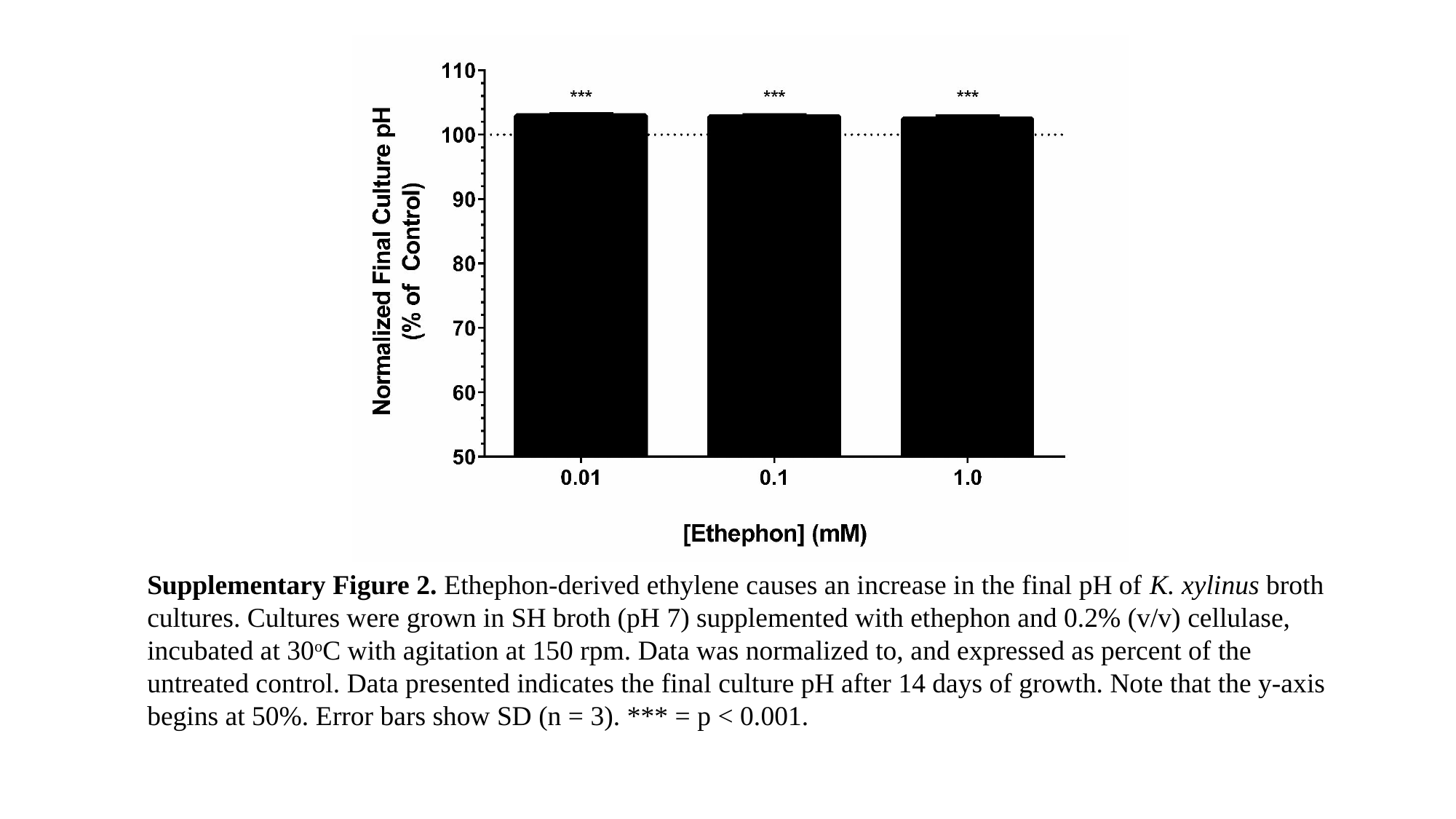

Supplementary Figure 2. Ethephon-derived ethylene causes an increase in the final pH of K. xylinus broth cultures. Cultures were grown in SH broth (pH 7) supplemented with ethephon and 0.2% (v/v) cellulase, incubated at 30oC with agitation at 150 rpm. Data was normalized to, and expressed as percent of the untreated control. Data presented indicates the final culture pH after 14 days of growth. Note that the y-axis begins at 50%. Error bars show SD (n = 3). *** = p < 0.001.

## Slide 5
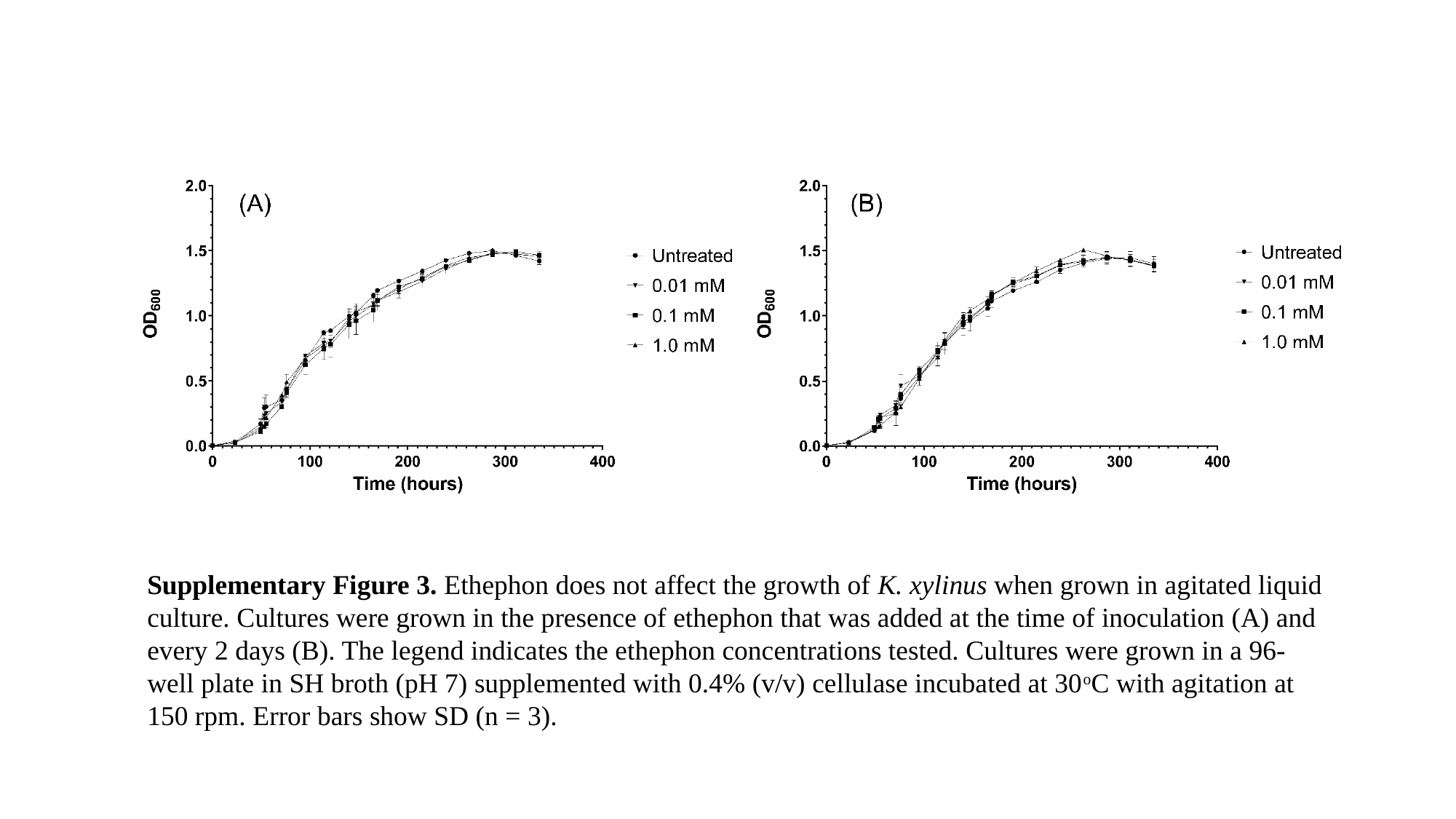

Supplementary Figure 3. Ethephon does not affect the growth of K. xylinus when grown in agitated liquid culture. Cultures were grown in the presence of ethephon that was added at the time of inoculation (A) and every 2 days (B). The legend indicates the ethephon concentrations tested. Cultures were grown in a 96-well plate in SH broth (pH 7) supplemented with 0.4% (v/v) cellulase incubated at 30oC with agitation at 150 rpm. Error bars show SD (n = 3).

## Slide 6
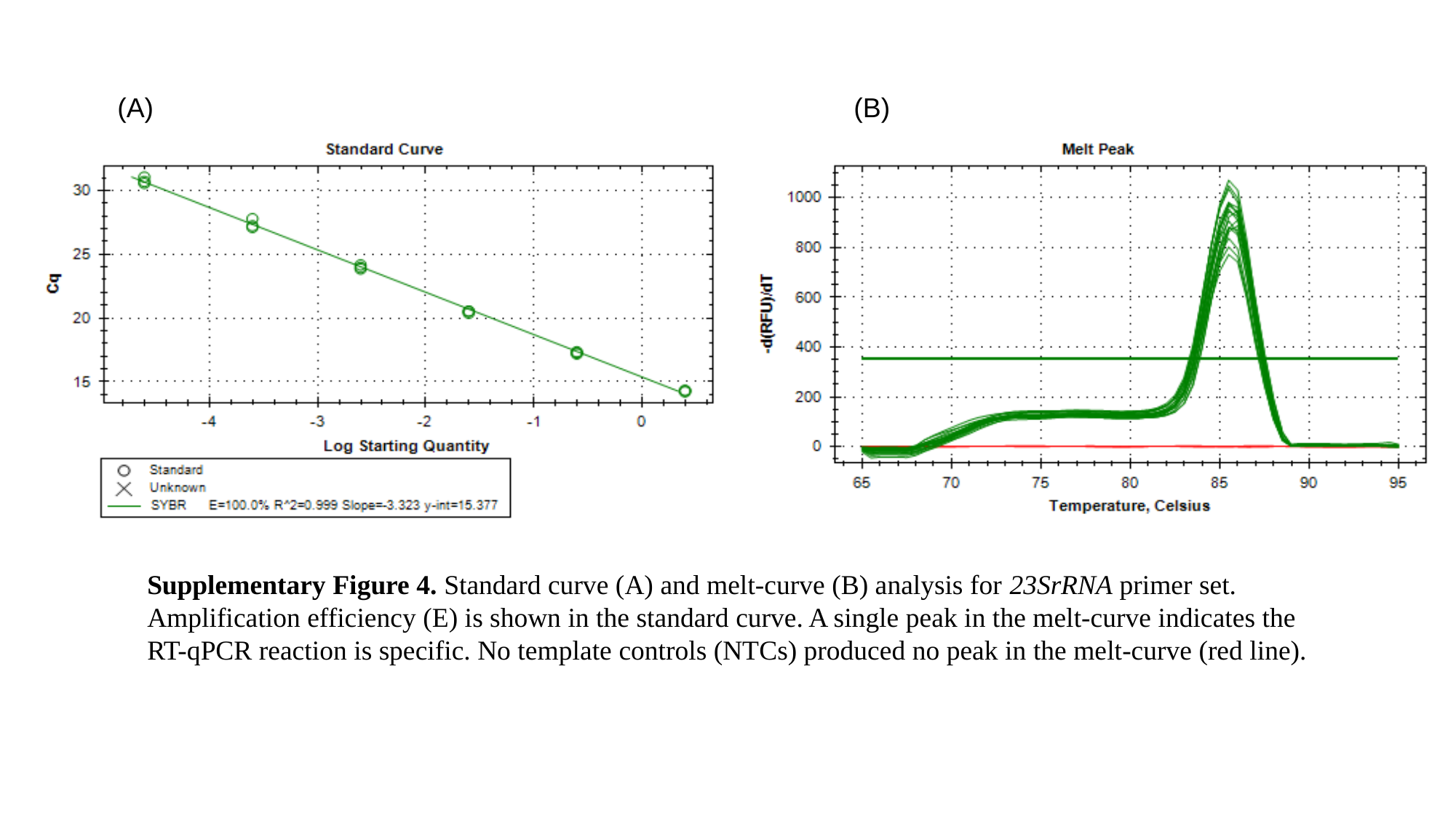

(A)
(B)
Supplementary Figure 4. Standard curve (A) and melt-curve (B) analysis for 23SrRNA primer set. Amplification efficiency (E) is shown in the standard curve. A single peak in the melt-curve indicates the RT-qPCR reaction is specific. No template controls (NTCs) produced no peak in the melt-curve (red line).

## Slide 7
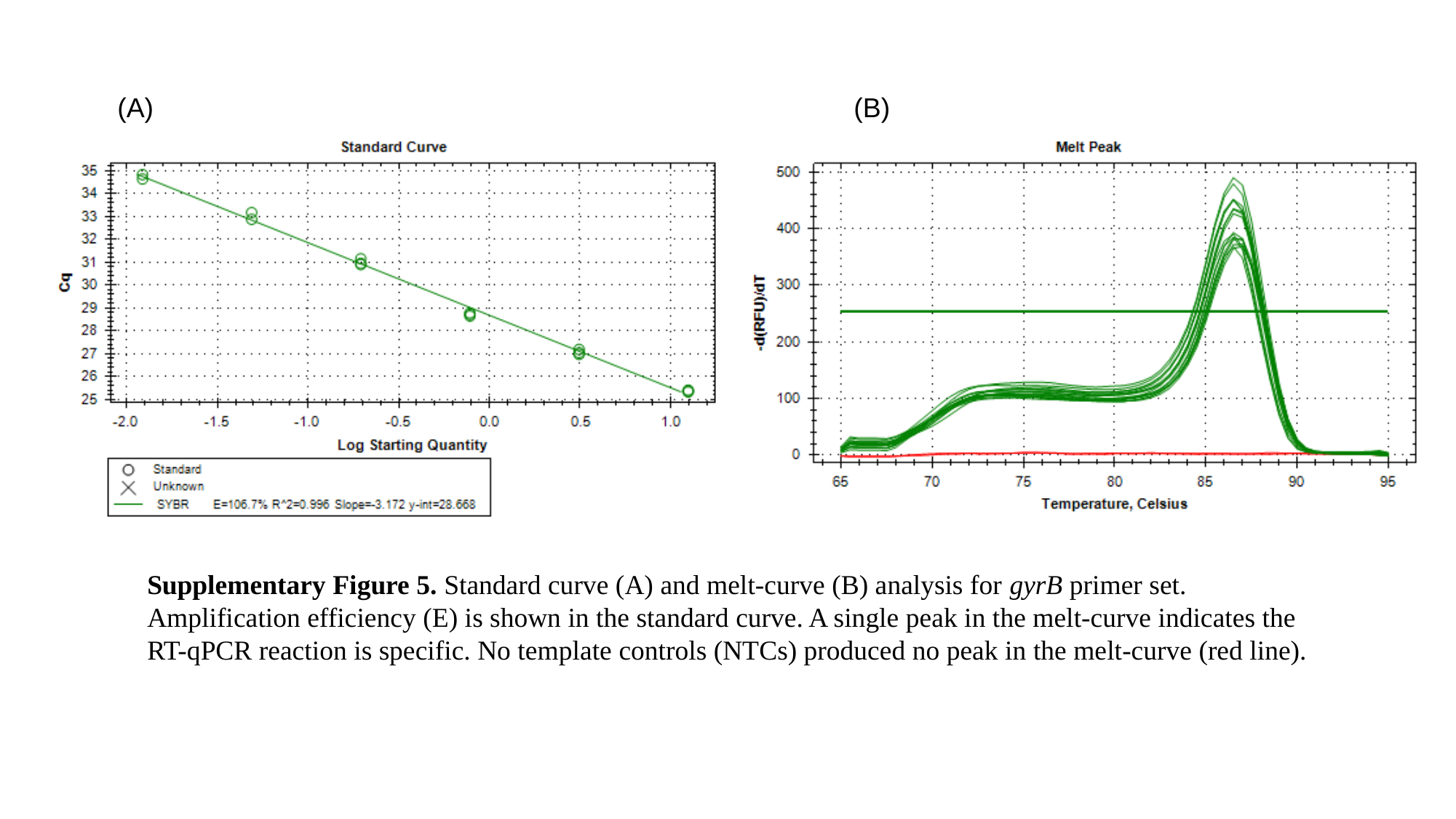

(A)
(B)
Supplementary Figure 5. Standard curve (A) and melt-curve (B) analysis for gyrB primer set. Amplification efficiency (E) is shown in the standard curve. A single peak in the melt-curve indicates the RT-qPCR reaction is specific. No template controls (NTCs) produced no peak in the melt-curve (red line).

## Slide 8
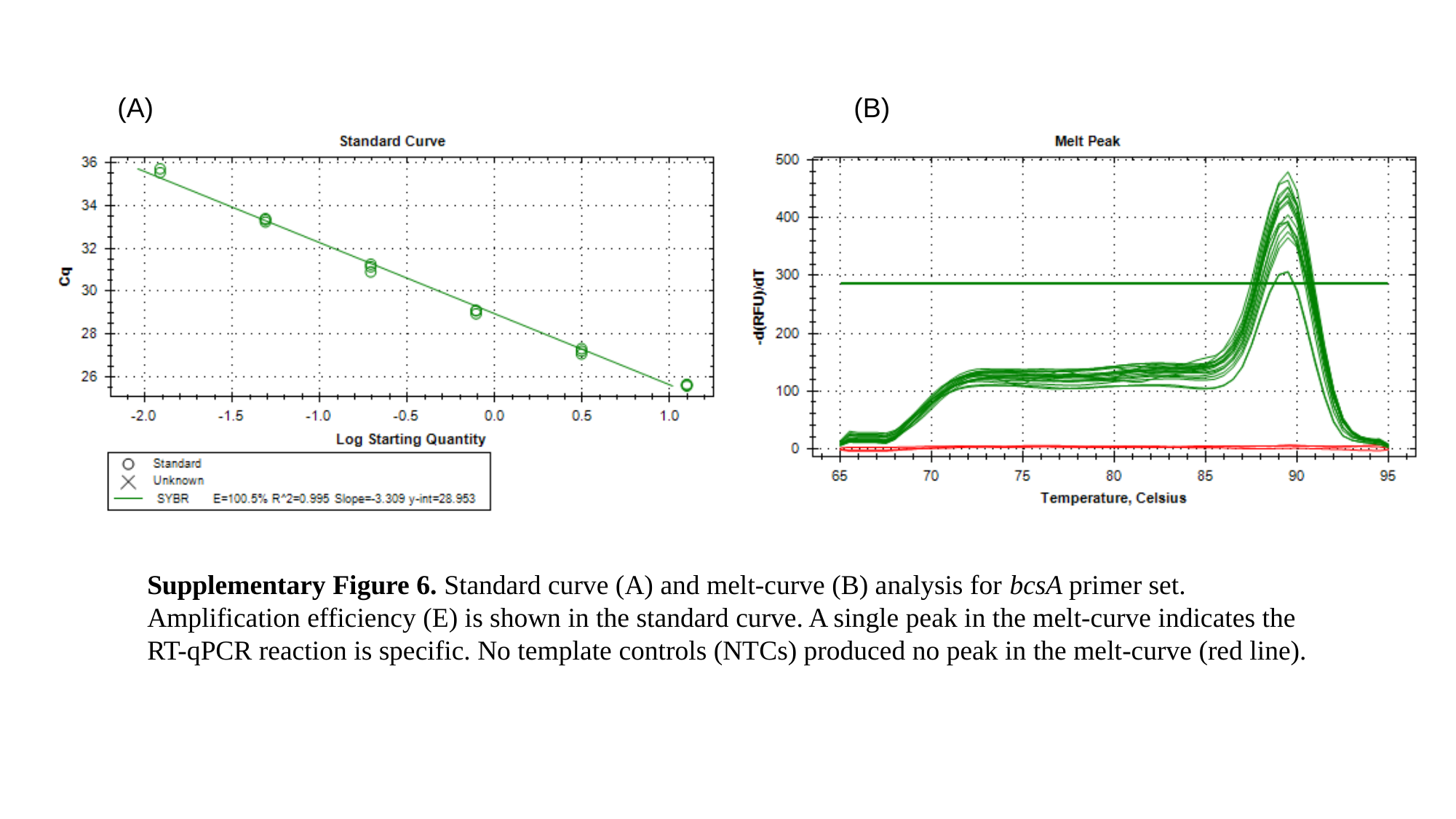

(A)
(B)
Supplementary Figure 6. Standard curve (A) and melt-curve (B) analysis for bcsA primer set. Amplification efficiency (E) is shown in the standard curve. A single peak in the melt-curve indicates the RT-qPCR reaction is specific. No template controls (NTCs) produced no peak in the melt-curve (red line).

## Slide 9
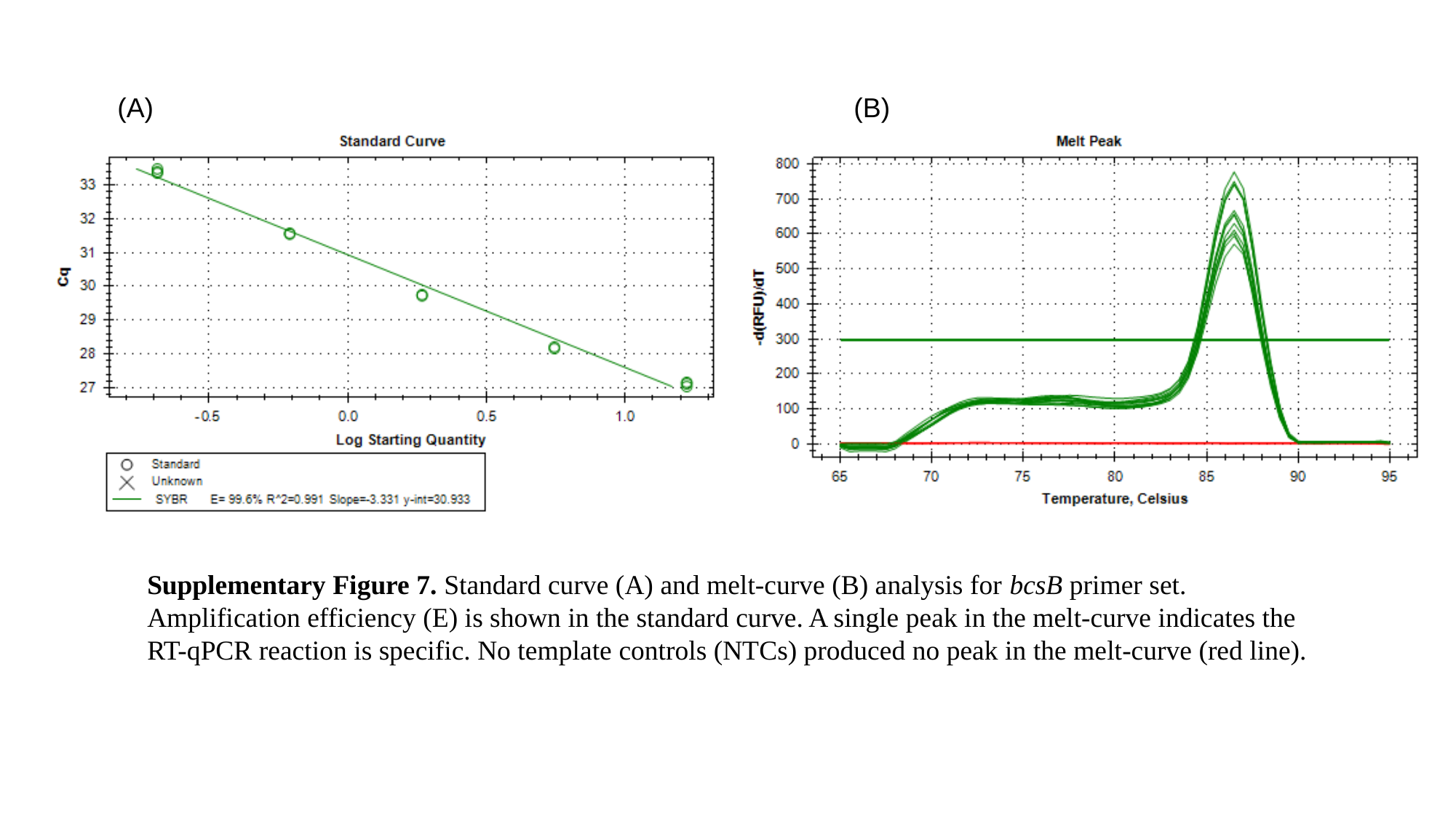

(A)
(B)
Supplementary Figure 7. Standard curve (A) and melt-curve (B) analysis for bcsB primer set. Amplification efficiency (E) is shown in the standard curve. A single peak in the melt-curve indicates the RT-qPCR reaction is specific. No template controls (NTCs) produced no peak in the melt-curve (red line).

## Slide 10
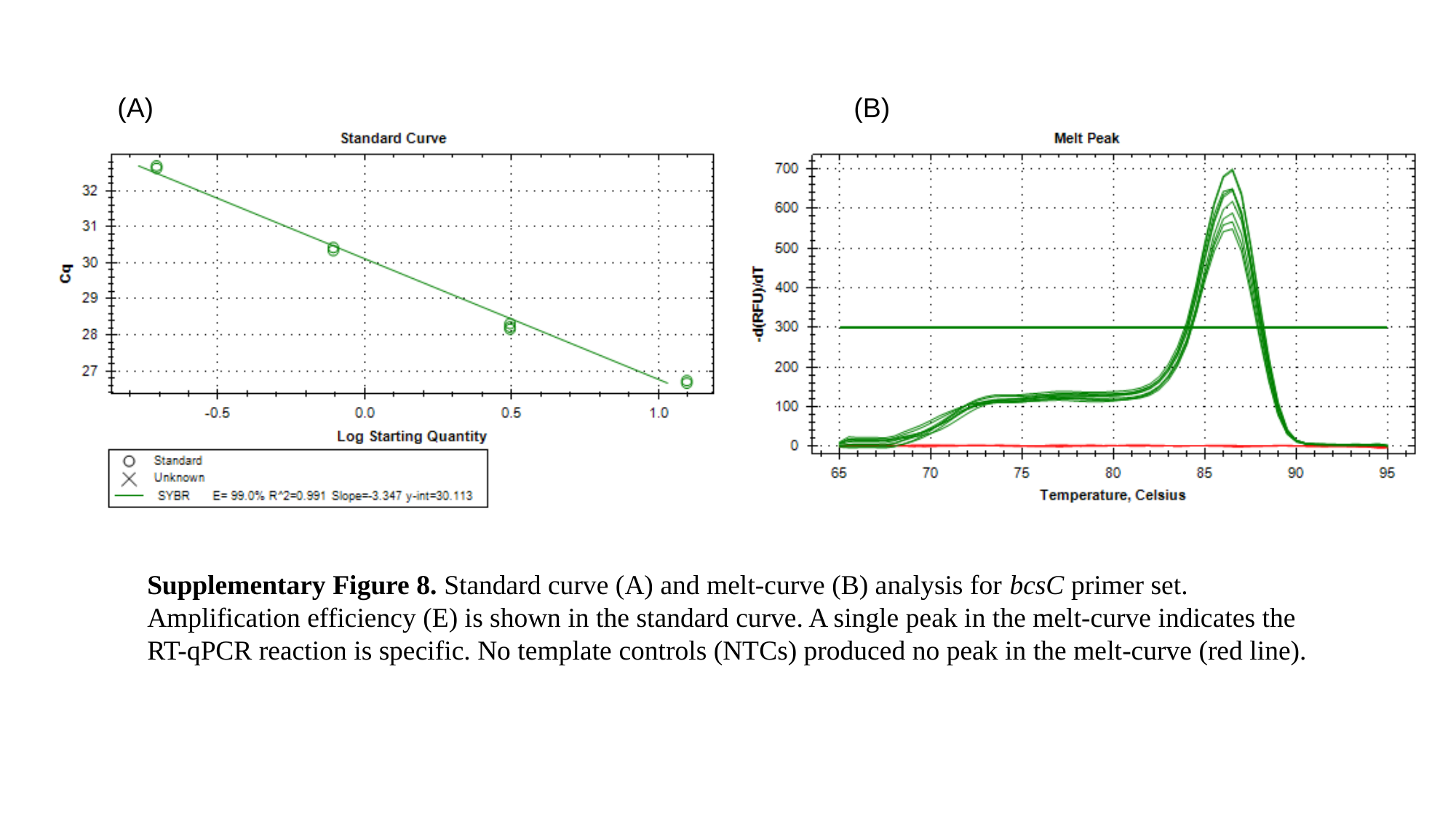

(A)
(B)
Supplementary Figure 8. Standard curve (A) and melt-curve (B) analysis for bcsC primer set. Amplification efficiency (E) is shown in the standard curve. A single peak in the melt-curve indicates the RT-qPCR reaction is specific. No template controls (NTCs) produced no peak in the melt-curve (red line).

## Slide 11
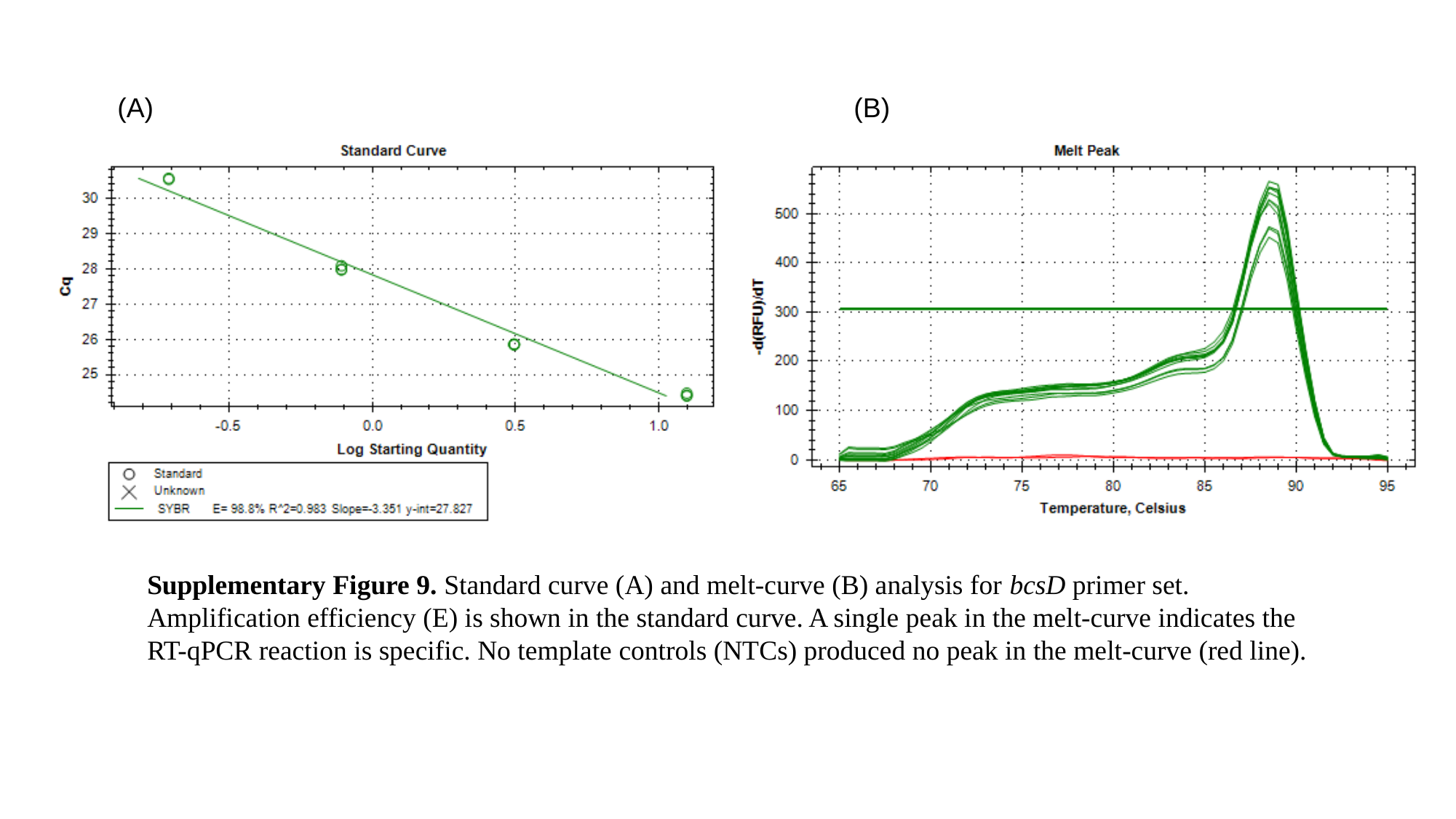

(A)
(B)
Supplementary Figure 9. Standard curve (A) and melt-curve (B) analysis for bcsD primer set. Amplification efficiency (E) is shown in the standard curve. A single peak in the melt-curve indicates the RT-qPCR reaction is specific. No template controls (NTCs) produced no peak in the melt-curve (red line).

## Slide 12
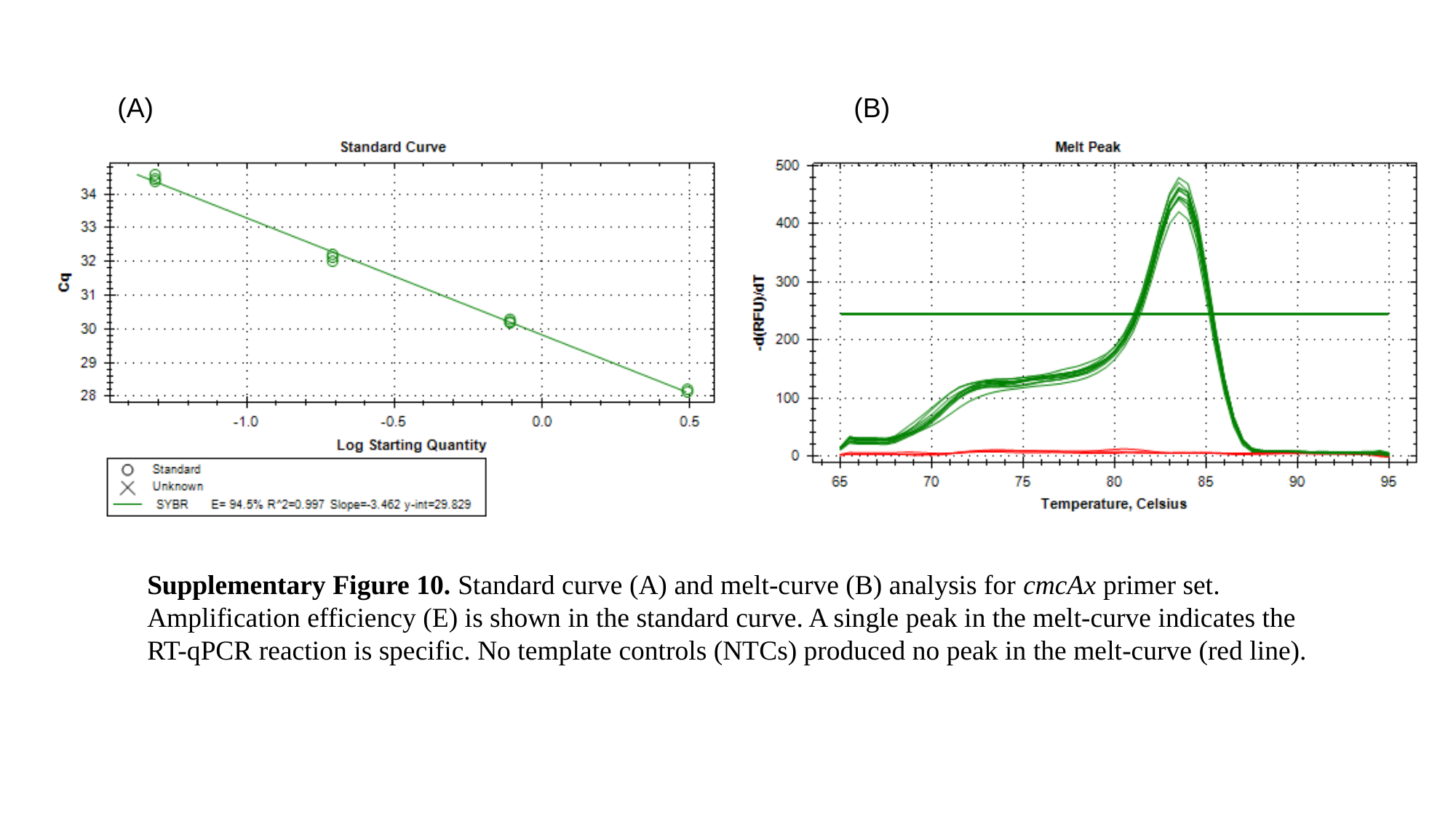

(A)
(B)
Supplementary Figure 10. Standard curve (A) and melt-curve (B) analysis for cmcAx primer set. Amplification efficiency (E) is shown in the standard curve. A single peak in the melt-curve indicates the RT-qPCR reaction is specific. No template controls (NTCs) produced no peak in the melt-curve (red line).

## Slide 13
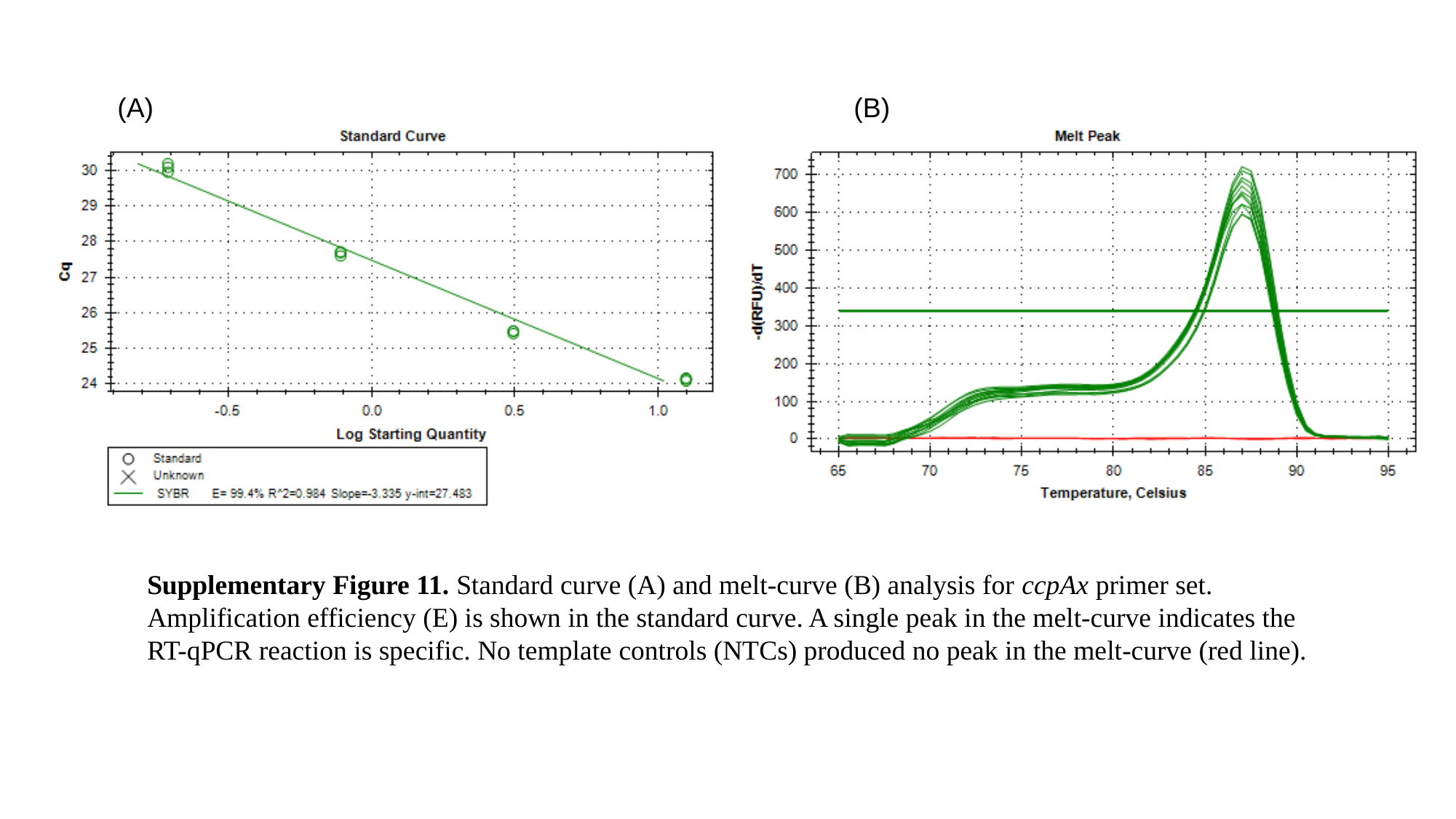

(A)
(B)
Supplementary Figure 11. Standard curve (A) and melt-curve (B) analysis for ccpAx primer set. Amplification efficiency (E) is shown in the standard curve. A single peak in the melt-curve indicates the RT-qPCR reaction is specific. No template controls (NTCs) produced no peak in the melt-curve (red line).

## Slide 14
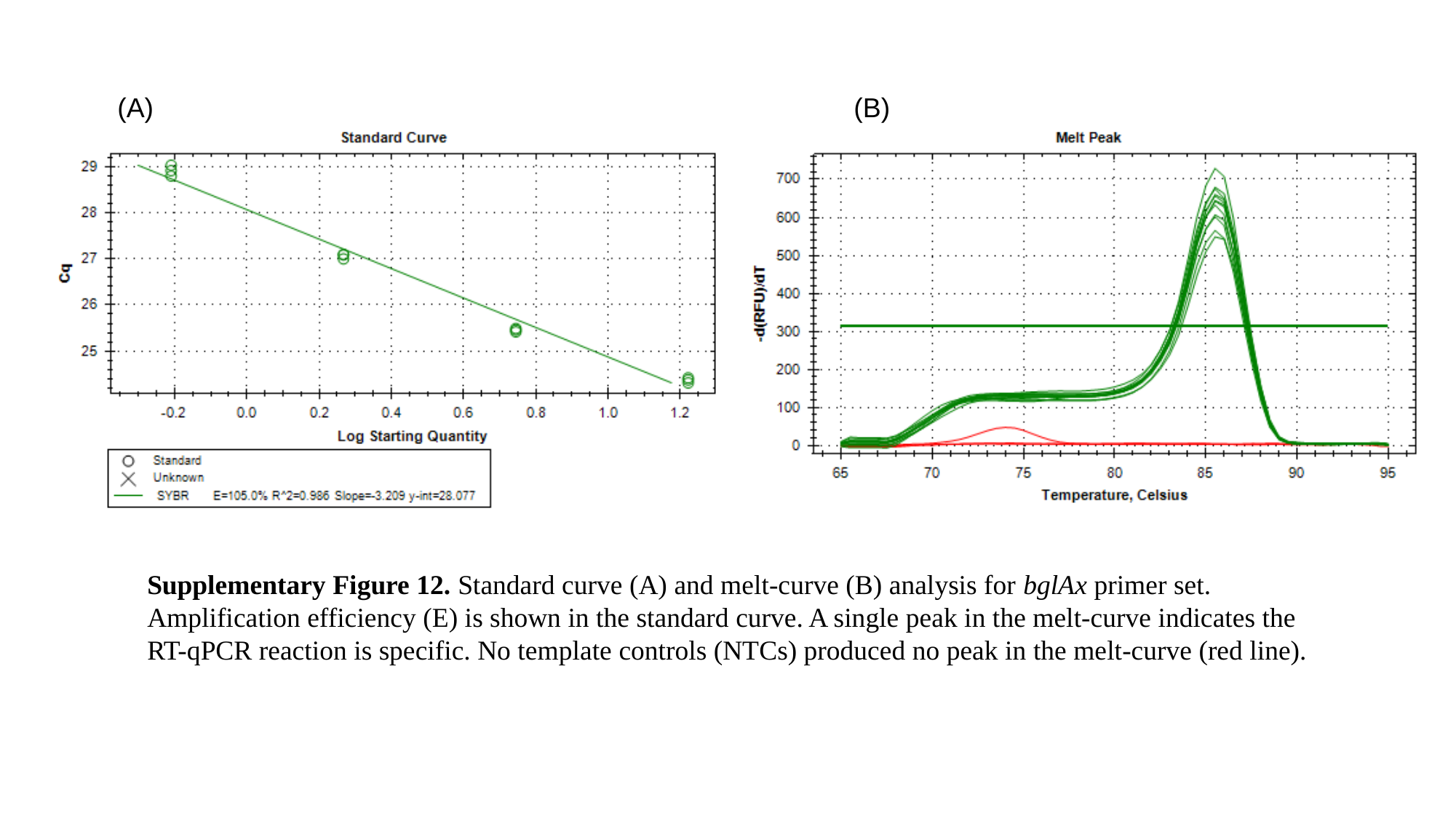

(A)
(B)
Supplementary Figure 12. Standard curve (A) and melt-curve (B) analysis for bglAx primer set. Amplification efficiency (E) is shown in the standard curve. A single peak in the melt-curve indicates the RT-qPCR reaction is specific. No template controls (NTCs) produced no peak in the melt-curve (red line).

## Slide 15
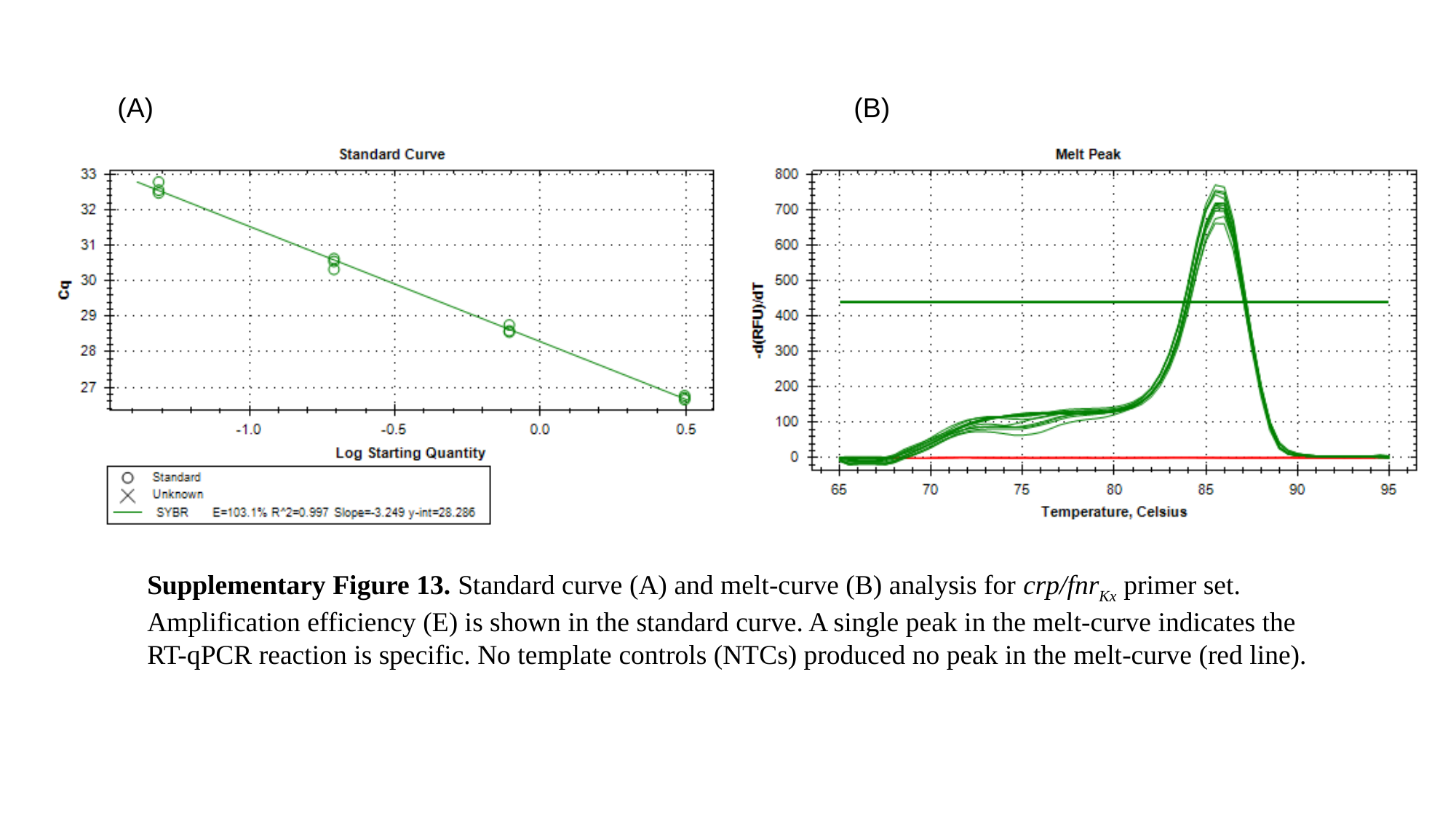

(A)
(B)
Supplementary Figure 13. Standard curve (A) and melt-curve (B) analysis for crp/fnrKx primer set. Amplification efficiency (E) is shown in the standard curve. A single peak in the melt-curve indicates the RT-qPCR reaction is specific. No template controls (NTCs) produced no peak in the melt-curve (red line).

## Slide 16
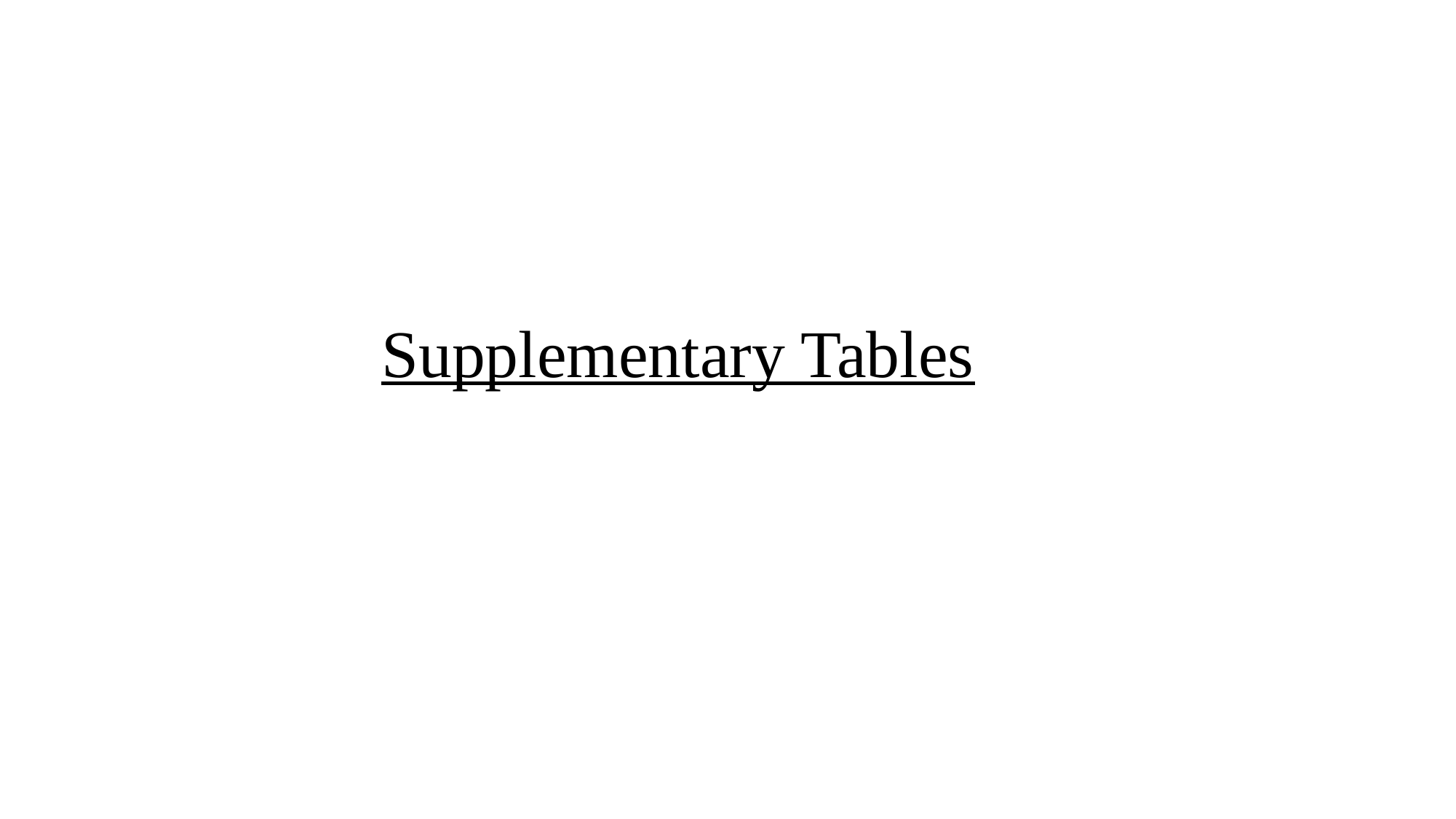

# Supplementary Tables

## Slide 17
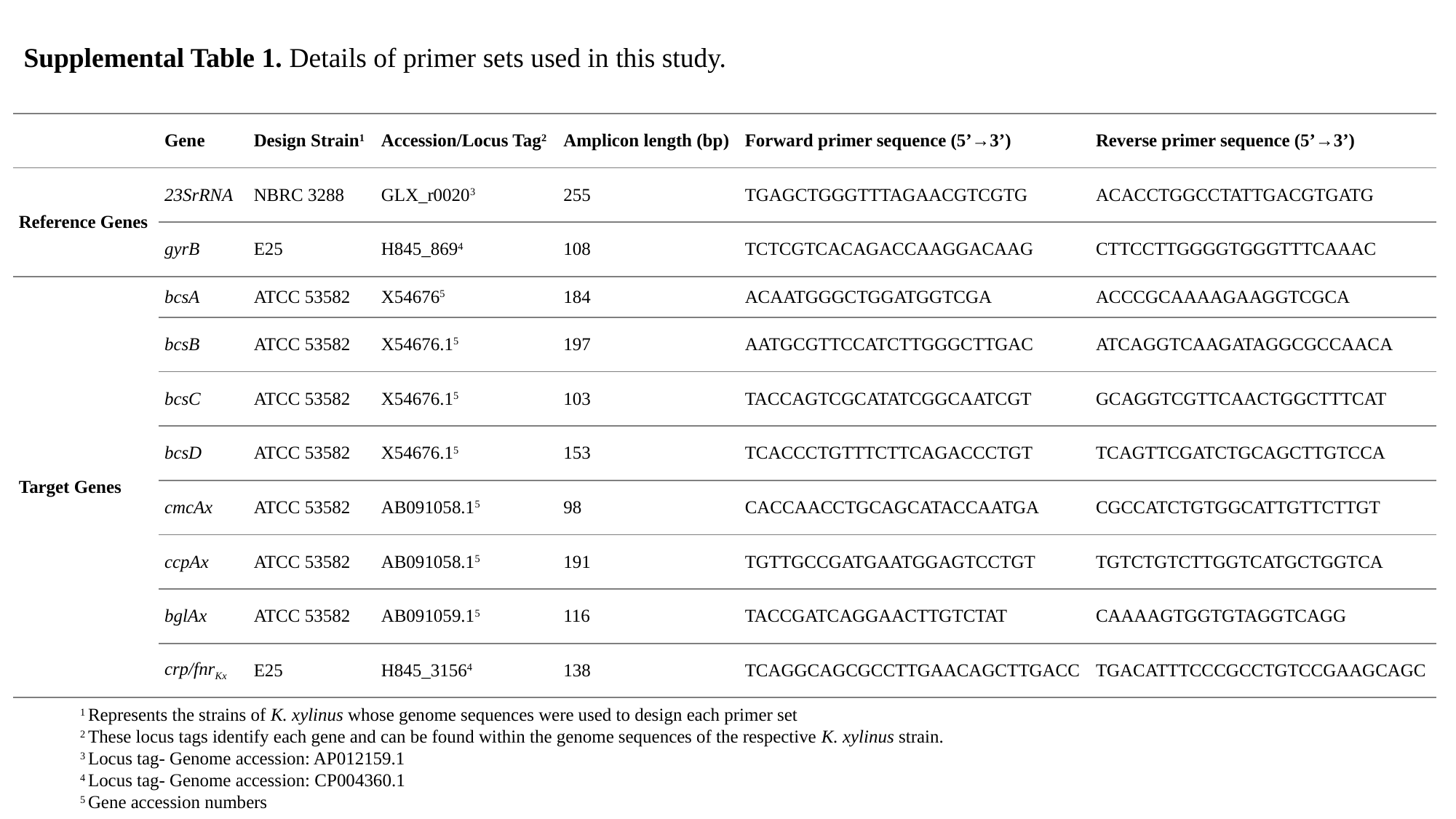

Supplemental Table 1. Details of primer sets used in this study.
| | Gene | Design Strain1 | Accession/Locus Tag2 | Amplicon length (bp) | Forward primer sequence (5’→3’) | Reverse primer sequence (5’→3’) |
| --- | --- | --- | --- | --- | --- | --- |
| Reference Genes | 23SrRNA | NBRC 3288 | GLX\_r00203 | 255 | TGAGCTGGGTTTAGAACGTCGTG | ACACCTGGCCTATTGACGTGATG |
| | gyrB | E25 | H845\_8694 | 108 | TCTCGTCACAGACCAAGGACAAG | CTTCCTTGGGGTGGGTTTCAAAC |
| Target Genes | bcsA | ATCC 53582 | X546765 | 184 | ACAATGGGCTGGATGGTCGA | ACCCGCAAAAGAAGGTCGCA |
| | bcsB | ATCC 53582 | X54676.15 | 197 | AATGCGTTCCATCTTGGGCTTGAC | ATCAGGTCAAGATAGGCGCCAACA |
| | bcsC | ATCC 53582 | X54676.15 | 103 | TACCAGTCGCATATCGGCAATCGT | GCAGGTCGTTCAACTGGCTTTCAT |
| | bcsD | ATCC 53582 | X54676.15 | 153 | TCACCCTGTTTCTTCAGACCCTGT | TCAGTTCGATCTGCAGCTTGTCCA |
| | cmcAx | ATCC 53582 | AB091058.15 | 98 | CACCAACCTGCAGCATACCAATGA | CGCCATCTGTGGCATTGTTCTTGT |
| | ccpAx | ATCC 53582 | AB091058.15 | 191 | TGTTGCCGATGAATGGAGTCCTGT | TGTCTGTCTTGGTCATGCTGGTCA |
| | bglAx | ATCC 53582 | AB091059.15 | 116 | TACCGATCAGGAACTTGTCTAT | CAAAAGTGGTGTAGGTCAGG |
| | crp/fnrKx | E25 | H845\_31564 | 138 | TCAGGCAGCGCCTTGAACAGCTTGACC | TGACATTTCCCGCCTGTCCGAAGCAGC |
1 Represents the strains of K. xylinus whose genome sequences were used to design each primer set
2 These locus tags identify each gene and can be found within the genome sequences of the respective K. xylinus strain.
3 Locus tag- Genome accession: AP012159.1
4 Locus tag- Genome accession: CP004360.1
5 Gene accession numbers

## Slide 18
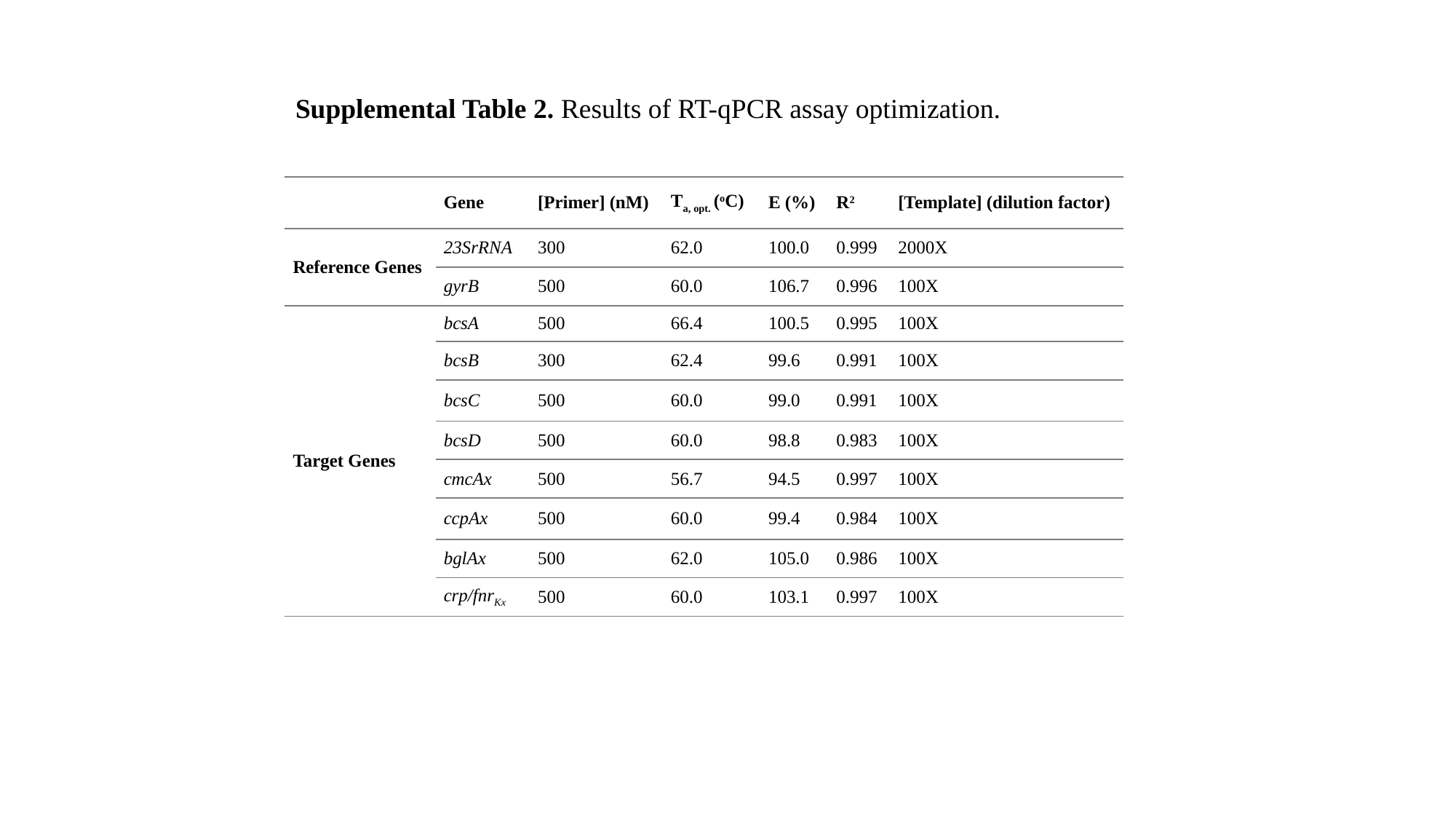

Supplemental Table 2. Results of RT-qPCR assay optimization.
| | Gene | [Primer] (nM) | Ta, opt. (oC) | E (%) | R2 | [Template] (dilution factor) |
| --- | --- | --- | --- | --- | --- | --- |
| Reference Genes | 23SrRNA | 300 | 62.0 | 100.0 | 0.999 | 2000X |
| | gyrB | 500 | 60.0 | 106.7 | 0.996 | 100X |
| Target Genes | bcsA | 500 | 66.4 | 100.5 | 0.995 | 100X |
| | bcsB | 300 | 62.4 | 99.6 | 0.991 | 100X |
| | bcsC | 500 | 60.0 | 99.0 | 0.991 | 100X |
| | bcsD | 500 | 60.0 | 98.8 | 0.983 | 100X |
| | cmcAx | 500 | 56.7 | 94.5 | 0.997 | 100X |
| | ccpAx | 500 | 60.0 | 99.4 | 0.984 | 100X |
| | bglAx | 500 | 62.0 | 105.0 | 0.986 | 100X |
| | crp/fnrKx | 500 | 60.0 | 103.1 | 0.997 | 100X |
